# G-Quadruplexes and Their Ligands: Biophysical Methods to Unravel G-Quadruplex/Ligand Interactions

**DOI:** 10.3390/ph14080769

**Published:** 2021-08-05

**Authors:** Tiago Santos, Gilmar F. Salgado, Eurico J. Cabrita, Carla Cruz

**Affiliations:** 1 CICS-UBI—Centro de Investigação em Ciências da Saúde, Universidade da Beira Interior, Av. Infante D. Henrique, 6200-506 Covilhã, Portugal; tiago.santos@fcsaude.ubi.pt; 2 ARNA Laboratory, Université de Bordeaux, Inserm U1212, CNRS UMR 5320, IECB, 33607 Pessac, France; gilmar.salgado@u-bordeaux.fr; 3 UCIBIO, REQUIMTE, Departamento de Química, Faculdade de Ciências e Tecnologia, Universidade Nova de Lisboa, 2829-516 Caparica, Portugal; ejc@fct.unl.pt; 4 Associate Laboratory i4HB—Institute for Health and Bioeconomy, NOVA School of Science and Technology, NOVA University Lisbon, 2819-516 Caparica, Portugal

**Keywords:** G-quadruplex, ligands, molecular interactions, biophysical methods

## Abstract

Progress in the design of G-quadruplex (G4) binding ligands relies on the availability of approaches that assess the binding mode and nature of the interactions between G4 forming sequences and their putative ligands. The experimental approaches used to characterize G4/ligand interactions can be categorized into structure-based methods (circular dichroism (CD), nuclear magnetic resonance (NMR) spectroscopy and X-ray crystallography), affinity and apparent affinity-based methods (surface plasmon resonance (SPR), isothermal titration calorimetry (ITC) and mass spectrometry (MS)), and high-throughput methods (fluorescence resonance energy transfer (FRET)-melting, G4-fluorescent intercalator displacement assay (G4-FID), affinity chromatography and microarrays. Each method has unique advantages and drawbacks, which makes it essential to select the ideal strategies for the biological question being addressed. The structural- and affinity and apparent affinity-based methods are in several cases complex and/or time-consuming and can be combined with fast and cheap high-throughput approaches to improve the design and development of new potential G4 ligands. In recent years, the joint use of these techniques permitted the discovery of a huge number of G4 ligands investigated for diagnostic and therapeutic purposes. Overall, this review article highlights in detail the most commonly used approaches to characterize the G4/ligand interactions, as well as the applications and types of information that can be obtained from the use of each technique.

## 1. Introduction

**Figure 1:**
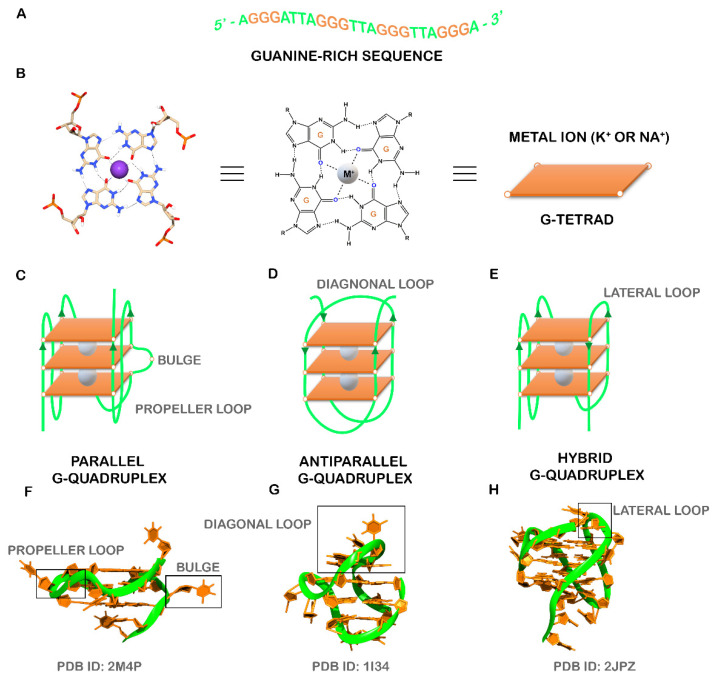
(**A**) Guanine-rich sequence with potential to form a three-tetrad G4. (**B**) Chemical structure of G-tetrad formed by the Hoogsteen hydrogen-bonded guanines and central cation (colored in gray) coordinated to oxygen atoms. Schematic representation of common unimolecular G4s based on the strand direction: (**C**) parallel, (**D**) antiparallel, and (**E**) hybrid. Representative PDB structures of (**F**) parallel (PDB ID: 2M4P), (**G**) antiparallel (PDB ID: 1I34) and (**H**) hybrid (2JPZ) G4 structures. The different loops (propeller, diagonal and lateral) and a bulge were also shown.

The human genome and transcriptome contain several guanine-rich sequences, which have stimulated considerable interest from researchers since the first reports of their being folded into non-classical structural motifs known as G-quadruplexes (G4s) [1,2,3] (Figure 1A). These structures are characterized by the presence of two or more stacks of four guanines organized in a coplanar manner [4]. Each set of four guanines forms a building block, usually called a G-tetrad, that are stabilized by Hoogsteen hydrogen base-pairing in physiological conditions, π–π interactions as well as in the presence of positively charged monovalent cations (usually K^+^ and Na^+^) (Figure 1B) [5]. G4s are highly polymorphic and can adopt a wide variety of structures based on strand molecularity and strand direction, as well as length and loop composition [6]. According to molecularity, the structures may be distinguished as intramolecular or intermolecular [6]. Considering the direction of the strands, G4 structures may be classified as parallel, antiparallel and hybrid (Figure 1C–H). The loops are generally divided into three main groups: propeller, lateral, and diagonal [6]. Recently, some structural studies demonstrated the formation of G4 structures with longer loop lengths and bulges, opening the framework for the development of novel diagnostic and therapeutic approaches based on those features [7,8].

Computational algorithms were developed to predict the location of specific G4 sequence motifs in the human genome [9,10]. Such predictors consisted of the general motif G_≥3_N_x_G_≥3_N_x_G_≥3_N_x_G_≥3_ and identified over 370,000 sequences with the potential to fold into G4 structures [11]. However, the early algorithms were not accurate and lacked the flexibility to accommodate divergences from the canonical pattern. In order to overcome these disadvantages, novel approaches were developed to compute the G4 propensity score by quantifying G-richness (reflecting the fraction of guanines in the sequence) and G-skewness (reflecting G/C asymmetry between the complementary nucleic acid strands) of a given sequence [12,13], or by summing the binding affinities of smaller regions within the G4 and penalizing with the destabilizing effect of loops [14]. Recently, new machine learning approaches were employed to map active G4s based on sequence features and trained using newly available genome wide mapping of G4s in vitro and in vivo [15,16].

**Figure 2:**
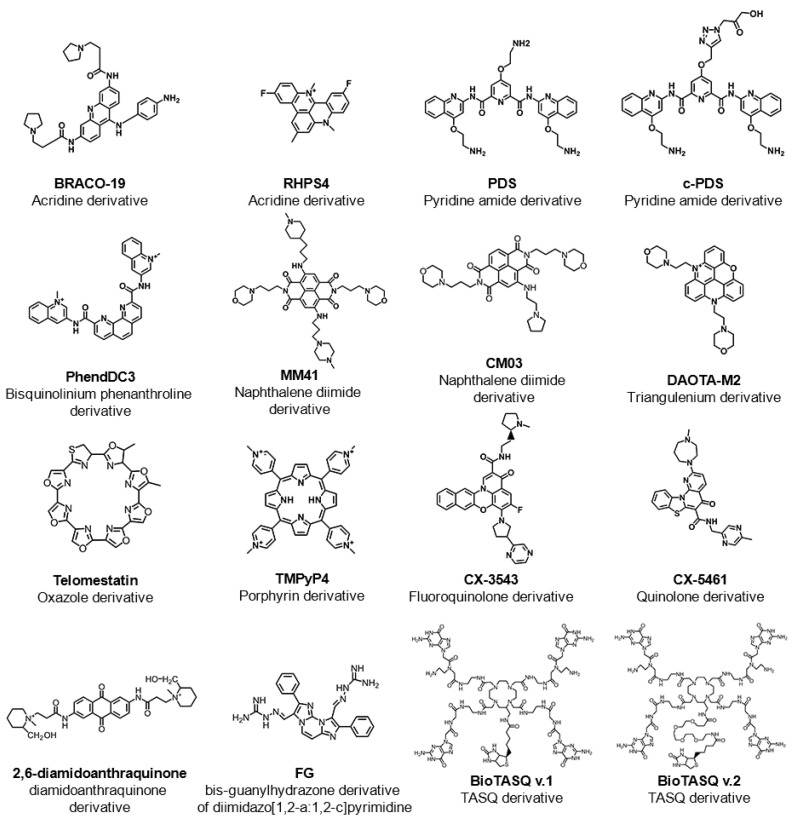
List of some examples of G4-interacting ligands mentioned in this review showing the common name of the ligand, chemical structure and family of the compound (chemical backbone).

In recent years, the development of high-throughput sequencing methods, such as G4-seq, has enabled the identification of over 716,000 DNA guanine-rich sequences across the human genome with the ability to fold into G4 structures in the presence of the well-known G4 ligand, pyridostatin (PDS) (Figure 2) [17]. PDS has an important role in next-generation sequencing (NGS), since it stabilizes G4s and induces polymerase stalling. Those DNA guanine-rich sequences are non-randomly distributed and are mainly located in clusters of immunoglobulin switch regions [18], telomeres [19] and promoter regions of oncogenes [20]. Several reports have described the formation of G4 structures within endogenous chromatin, and their ability to recruit transcription factors to promote active transcription [21,22,23,24,25,26,27,28]. The location of those G4 structures was revealed using an antibody-based G4 chromatin immunoprecipitation sequencing (G4 ChIP–seq) approach [21], and suggests that they play a crucial role in critical cellular processes such as DNA replication [29,30], DNA damage repair [26], transcription [22,23], translation [31] and epigenetic modifications [32]. By using G4 ChIP–seq, Hänsel-Hertsch et al. showed a reduction in the number of detected DNA G4s (10,000) in genome [21]. These results are not surprising since transient G4 structures strongly depend on chromatin relaxation and cell status [21]. Recently, an improved version of the G4-seq method was developed and makes available the G4 map of 12 different species [33].

RNA guanine-rich sequences came into the trends of research in the last few years due to their intrinsic features and strengths. RNA G4s are more compact, less hydrated, and more thermodynamically stable than their DNA counterparts [34]. Furthermore, the presence of the 2′-OH group in the ribose ring favors the parallel topology, making them more attractive as target molecules [34]. To date, using computational approaches, more than 1.1 million guanine-rich sequences h with the ability to fold into RNA G4 have been identified [35]. RNA G4s were shown to exist in human cells by using the specific G4 antibody BG4 [36] and, in the same way as DNA G4s, those sequences are non-randomly distributed in the transcriptome [37]. Those sequences are mainly located in both 5′ and 3′UTR, as well as at the splicing junction of mRNA and noncoding RNAs, being of utmost importance in regulatory post-transcriptional mechanisms [37]. In the last few years, several reports have highlighted the importance of G4s in the transcriptome by employing G4 sequencing high-throughput approaches [38,39,40,41]. rG4-seq was initially applied to map G4s in RNA extracted from HeLa cells [38] and later to plants [40] and bacteria [41]. G4RP-seq was also used to characterize the G4 transcriptomic landscape in vivo [39]. Yang et al. developed a biotinylated template-assembled synthetic G-quartet (TASQ) derivative (BioTASQ v.1) (Figure 2) and captured G4 RNAs from breast cancer cells in log-phase growth, followed by target identification by sequencing [39]. The effect of BRACO-19 and RHPS4 (Figure 2) treatment was also evaluated [39]. They found that those ligands can change the G4 transcriptome in a more remarkable way in long non-coding RNAs [39]. More recently, the same research group developed a new BioTASQ prototype that they called BioTASQ v.2 (Figure 2) and performed an in-depth study of both ligands [42]. Those studies are of utmost importance and revealed the strong relevance that G4 ligands could have in cell biology.

Therefore, the location of G4s at both DNA and RNA levels suggests an active role in the development of diseases such as cancer and neurological disorders [43]. Several pieces of evidence suggest that G4s play an important role in promoting genomic instability by triggering DNA damage [44,45,46]. The G4 ligand PDS induces DNA damage as shown by the formation of γH2AX foci, a marker of double-stranded DNA breakage (DSB) [47]. Furthermore, ChIP-seq has shown that PDS accumulates at genes containing clusters of G4 structures and that accumulation is transcription-dependent [47]. Recently, De Magis et al. showed that the G4 ligands PDS, BRACO-19 and bis-guanylhydrazone derivative of diimidazo(1,2-a:1,2-c)pyrimidine 1 (FG) (Figure 2) induced the formation of R-loops, another noncanonical secondary of a DNA:RNA hybrid compatible with the formation of a G4, and promote DNA damage as a consequence of that formation [44]. They also found that the mechanism of genome instability and cell killing by G4 ligands was particularly efficient in *BRCA2*-depleted cancer cells [44]. This study could open up new possibilities of investigation and lead to the development of new anticancer approaches.

Although the G4s present in eukaryotic species have been extensively studied, their presence in bacteria and viruses has only attracted attention in the last few years [48,49,50,51,52]. In bacteria, G4s are found in regulatory regions that play important functions in replication, radioresistance, antigenic variation and latency [51]. G4s in viruses have important regulatory roles in key viral steps [53]. Recent studies have demonstrated the formation and function of G4s in pathogens responsible for serious diseases. Among them are *Mycobacterium tuberculosis* [54], *Pseudomonas aeruginosa* [41], Human Papilloma Virus (HPV) [55], Human Immunodeficiency Virus (HIV) [53] and SARS-CoV-2 [56].

Therefore, the recognition of the biological significance of G4s has promoted the research and development of ligands that interact with G4s and regulate their structure and function. The most well-known G4 ligands were initially developed to target DNA G4s, but many of them have also been employed to target of RNA G4s [57]. Despite some significant progress in the field, the main challenge remains on the trade-off between affinity and selectivity, which could be achieved with the full characterization of G4/ligand interactions. Since the discovery of the first G4 ligands (disubstituted amidoanthraquinones) (Figure 2) [58], methods such as circular dichroism, surface plasmon resonance, isothermal titration calorimetry, mass spectrometry, nuclear magnetic resonance and X-ray crystallography have been used to characterize the molecular interactions of the G4/ligand pair. However, despite the utility of those methods, they are in general, time-consuming and/or costly for the first screening of G4/ligand interactions. Following the general tendency, high-throughput approaches such as FRET-melting, G4-Fluorescence intercalator displacement (G4-FID), affinity chromatography and microarrays have emerged as rapid and efficient methods to detect the binding and interaction of ligands with their G4 targets.

Overall, this review describes the most well-known G4 ligands and highlights the importance of the most recently developed experimental methods for characterizing G4/ligand complex interactions.

## 2. Overview of G4-Interacting Ligands

The increasing evidence that DNA and RNA G4s can regulate a variety of physiological functions in cancer and neurological disorders has encouraged the design and development of new G4-interacting ligands that may act as therapeutic agents [59]. However, besides acting as potential therapeutic agents, ligands can be utilized as molecular agents in biosensing and bioimaging for diagnostic purposes [60].

To date, a significant number of ligands targeting G4s have been investigated and most of them have been deposited in the G4 Ligands Database 2.1 (http://www.g4ldb.com/ (accessed on 3 August 2021)) [61]. Some G4 ligands are highlighted in Figure 2. Most of these well-known G4 ligands share common structural features such as an aromatic core, which permits π−π stacking interactions with planar G-tetrads, and one or more positive moieties that may interact with DNA or RNA backbone phosphate groups in grooves and loops [62]. The dissociation constant (*K*_D_) that measures to the binding affinity is a key parameter of affinity in G4/ligand interaction and for most of the G4/ligand complexes is lower than 10^−6^ M [62]. However, in addition to affinity and stabilization, the G4 ligands must display selectivity between G4 and duplexes’ topologies. It is worth noting that some of the most well-known G4 ligands, such as BRACO-19 and TMPyP4 (Figure 2), display low selectivity to G4 structures over duplex DNA [63,64]. It was recently proposed that structure-activity relationship studies could significantly improve the physicochemical properties of ligands and enable the optimum trade-off between affinity and selectivity [65]. A recent review by Asamitsu et al. describes in great detail the ligand design and development to acquire specificity and selectivity without compromising affinity [66]. However, there is a significant lack of knowledge of the binding of ligands to G4s when they are embedded with duplex DNA or mRNA, and in chromatin contexts [67].

The early evidence that G4s can be found at the end of telomeres and on oncogene promoter regions led to much attention in the G4 field focusing on developing DNA G4 ligands [66] against cancer. Indeed, because of the low pharmacological properties, which include poor selectivity, no G4 ligand has advanced beyond Phase II trials. Until now, the only ligand that reached this phase was the fluoroquinolone derivative, quarfloxin (CX-3543), which binds to G4s located in ribosomal DNA (rDNA) and disrupts nucleolin interaction [68]. CX-5461 is another G4 ligand that is currently at advanced phase I clinical trials for patients with *BRCA1/2* deficient tumors [69]. Recently, Masud et al. demonstrated that inhibition of the critical member of the DNA damage response, *UBE2N*, acted synergistically with CX-5461 increasing cell toxicity [70]. Further, this compound has shown the potential to suppress pulmonary arterial hypertension and associated vascular remodeling and pulmonary inflammation by inhibiting the RNA polymerase I [71]. For those reasons, the unique chemical qualities of RNA G4s, together with their location in key regions of the human transcriptome, have spurred the design and development of specific and selective RNA G4 ligands. Most of those ligands have been transposed from DNA G4s and others have been optimized with functional groups to obtain better affinity and selectivity. In this section, we describe some of the compounds that are lastly reported as DNA or RNA G4 ligands, as well as their binding mode and interactions.

### 2.1. DNA G4-Interacting Ligands

As previously mentioned, since guanine-rich sequences of telomeric ends and oncogene promoter regions have been considered attractive targets for cancer therapeutics, a significant number of ligands targeting DNA G4s have been reported [20,72].

**Figure 3:**
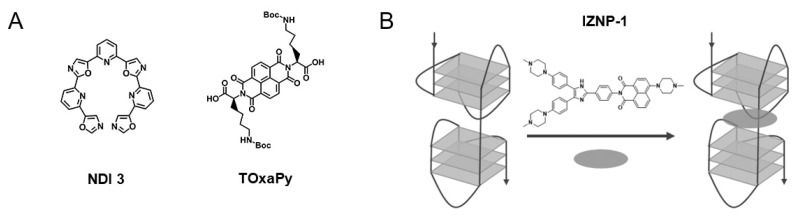
Examples of selective DNA G4 ligands. (**A**) Chemical structures and family of the ligands TOxaPy and NDI 3. (**B**) Chemical structure of IZNP-1 and proposed binding model of the intercalation of IZNP-1 into two telomeric G4 units. Adapted from ref. [77].

Telomeres are nucleoprotein structures that are crucial in genome stability and cell growth due to protecting chromosome ends. In humans, telomeres consist of tandem repeats of the highly conserved repetitive sequence d(TTAGGG), ending in a shorter G-rich single-stranded 3′-overhang. Those tandem repeats can form G4 structures that could impact telomerase activity. BRACO-19 is a 3,6,9-trisubstituted acridine derivative and one of the first G4 ligands developed with specific telomerase inhibitory activity by targeting G4s in telomeres [73]. Despite promising in vitro and in vivo anticancer activity, its potential use in clinical settings was hindered by its low selectivity toward DNA G4s over duplex [73]. Recently, the binding modes of BRACO-19 toward different telomeric DNA G4 topologies were tested by molecular dynamics simulations with an explicit solvent and revealed an end-stacking mode for the parallel G4s, bottom stacking mode for the antiparallel G4s, and top stacking mode for hybrid G4s [74]. The lack of preferential binding selectivity of BRACO-19 toward G4s over duplex could be explained by the similar binding affinity of groove binding mode for both G4 and duplex structures [74]. In order to tackle the issue of selectivity without compromising binding affinity, one approach that has been used is the use of loops and grooves with the ability to establish different interactions in distinct environments. The pyridine-oxazole derivative TOxaPy (Figure 3A) shows preferential binding to telomeric G4s with antiparallel topology over telomeric G4s with parallel topology [75]. The results also confirmed the much stronger binding affinity of the ligand in Na^+^ than in K^+^. The results in Na^+^ showed a stoichiometry of 1:1 and *K*_D_ = 2×10^−7^ M^−1^. Further evaluation of the interactions by molecular docking suggests a specific groove bind. The naphthalene diimide derivative NDI 3 (Figure 3A) was developed as a ligand with specificity for *c-KIT2* G4, and the preference for this interaction possibly relies on the specific contact with the loops or grooves, which confer binding preferences [76]. Another approach to gain selectivity is the use of G4 ligands that permit favorable discrimination of dimeric G4 forms from monomeric ones. A new triaryl-substituted imidazole derivative called IZNP-1 (Figure 3B) was reported and through several biophysical and in vitro techniques is was proven that the ligand stabilizes telomeric G4s specifically [77]. In addition, the binding mode was unveiled and revealed intercalation of the ligand into the pocket between the two G4 units (Figure 3B) [77].

**Figure 4:**
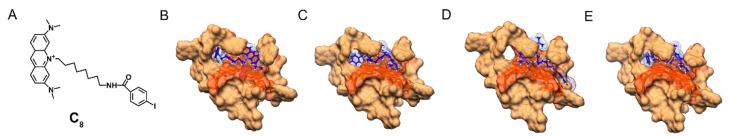
(**A**) Chemical structure of the acridine derivative C_8_. (**B**–**E**) Computational models of complex C_8_/*KRAS*22-RT generated by molecular docking after energy minimization. The figures (**B**–**E**) depicted conformers obtained with different binding free energies, −9.32, −7.24, −9.17 and −8.40 kcal/mol, respectively. G4 structure is depicted as a light orange surface with the 5′ tetrad highlighted in darker orange. C_8_ is represented in blue. Adapted from ref. [81].

Previous studies support the evidence that G4s located in promoter regions of *hTERT*, *c-MYC*, *BCL2*, *KRAS*, *HSP90* and *VEGF*, can serve as potential targets in cancer therapeutics [78]. Most of the G4 ligands used to hinder telomerase activity were also investigated for their ability to block the transcriptional activity of polymerase in promoter regions of oncogenes [78]. Besides those ligands, the naphthalene diimide derivatives, MM41 and CM03 (Figure 2), are binders and stabilizers of G4s in both telomeric ends and oncogene promoter regions (*HSP90, BCL2 and KRAS*), and exert a noteworthy antiproliferative effect in pancreatic cancer cells [79,80]. Furthermore, the computational and experimental approaches revealed that pyrrolidino side chains interact with G4s mainly through groove binding using electrostatic interactions. The acridine orange derivative C_8_ (Figure 4) was recently investigated for its ability to bind and strongly stabilize G4 structures in the *KRAS* oncogene promoter [81]. The NMR and docking studies demonstrated that ligand interacts with 22-mer *KRAS* 22RT G4 via π−π stacking and groove/loop interactions [81]. In addition, the acridine core permits end-stacking interactions with external G-tetrads, while alkylamide side chains establish contact points with grooves/loops. Interestingly, the positively charged nitrogen group may occupy the cavity, usually occupied by one of the three central ions through electrostatic interactions. The computational studies using NOE distance restraints revealed details about the ligand structural features in the interaction with *KRAS*-22RT G4 (Figure 4).

Nevertheless, the binding modes of other well-known G4 ligands such as telomestatin [82,83], RHPS4 [84,85], and naphthalene diimide derivatives [86,87] (Figure 2) are well-characterized by several computational and experimental approaches.

**Figure 5:**
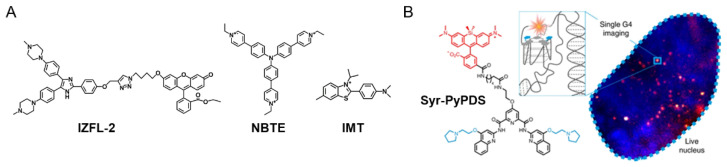
Fluorescent G4 probes. (**A**) Common name and chemical structures of IZFL-2, NBTE and IMT. (**B**) Chemical structure of SiR-PyPDS and single-molecule fluorescence imaging of G4s in living cells using the fluorescent probe SiR-PyPDS. Adapted with permission from ref. [97] Copyright 2020 Springer Nature.

Meanwhile, at the same time that G4 ligands have been used for therapeutic purposes, a range of G4 optical probes, suitable for diagnostic purposes, have been developed to recognize G4s selectively [60]. Hu et al. developed a specific fluorescent G4 probe that distinguishes *c-MYC* G4 from other G4 structures [88]. In addition, the authors described the binding model of IZFL-2 (Figure 5A), which is mainly based on π–π stacking and additional hydrogen bonding interactions [88]. Further, several reviews report G4 specific molecules endowed with fluorescence properties. These molecules represent a variety of structural scaffolds, a mechanism of G4-recognition and fluorescence signal transduction. The G4 selectivity and in vivo imaging potential of these molecules place them uniquely as G4-theranostic agents in the predominantly cancer therapeutic context of G4-selective ligands. Moreover, several probes were described to study the function and mechanism of G4 formation in mammalian cells [60,89,90,91,92]. Shivalingam et al. also reported a new G4 optical probe, DAOTA-M2 (Figure 2), which localizes and interacts with G4s [93]. Later, the binding mechanism of the ligand toward *c-MYC* G4 was thoroughly investigated [94]. Their study reveals that the DAOTA-M2 polyaromatic core establishes stacking interactions with external G-tetrads of the *c-MYC* G4. In fact, a wide range of DNA G4 optical probes (e.g., NBTE [95], IMT [96] and SiR-PyPDS [97]) (Figure 5) are increasingly being developed and characterized in recent years. The tripodal cationic fluorescent probe NBTE detected a percentage of G4 DNA in live cells and found 4-fold G4 DNA in cancer cells than in normal cells, suggesting the potential applications of this probe in cancer cell detection [95]. The benzothiazole derivative IMT is a fluorescent probe, reported by Zhang et al., that tracked DNA G4 changes at different points in cell progression and aphidicolin and hydroxyurea treatment, revealing more information on the roles of DNA G4s in biological systems [96].

Another example of a G4-specific fluorescent probe is SiR-PyPDS, which enables single-molecule and real-time detection of individual G4 structures in living cells without perturbing G4 formation and dynamics (Figure 5B) [97]. The authors also demonstrated that G4 formation in live cells is cell-cycle-dependent and disrupted by chemical inhibition of transcription and replication [97].

### 2.2. RNA G4-Interacting Ligands

Despite a growing number of RNA G4s being identified and characterized, reports on their interaction with G4 ligands are less common. Nevertheless, other RNA secondary structures have been extensively studied for therapeutic and diagnostic purposes [37]. Since RNA G4s are non-randomly distributed in transcriptome and present in several important genes and regulatory regions, designing G4 ligands that stabilize or destabilize such structures is seen as an attractive therapeutic and diagnostic strategy for various diseases such as cancer and neurological disorders [37]. The destabilization of G4 structures by G4 ligands is less commonly described; however, in recent years some studies have been reported for RNA G4s. For instance, some studies involving TMPyP4 have reported that the ligand destabilizes RNA G4s [98,99,100,101,102]. Despite this intriguing observation by different research groups, the mechanism of the unfolding of RNA G4s by TMPyP4 is still unknown.

Until recently, most RNA G4 structures were thought to conform in relatively simple RNA G4 structures, in which guanines stacking within the G4 would also be contiguous in sequence (e.g., four successive guanine trinucleotide tracts separated by loop nucleotides) [34]. Recently, crystallographic and NMR structure determinations of some in vitro selected RNA aptamers have revealed RNA G4 structures of unprecedented complexity [103]. Indeed, Sc1 and spiegelmer aptamers have nucleotides in syn conformation, locally inverted strand polarity, and nucleotide quartets that are not all-G. Common to these new structures, the sequences folding into G4s do not conform to the requirement that guanine stacks arise from consecutive nucleotides [103]. On the other hand, the G4 ligands design and development that provides recognition and selectivity exclusively for RNA has been laborious. In fact, most of the G4 ligands that have been originally designed and developed for DNA G4s have been later investigated to target RNA G4s. An interesting review by Tao et al. describes in detail the recent advances in the development of ligands toward RNA G4 structures [104].

**Figure 6:**
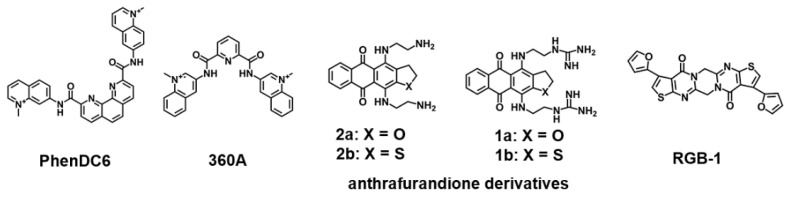
RNA G4-interacting ligands mentioned in this review showing the common name of the ligand and chemical structure.

Several pieces of evidence have pointed out that, similar to the DNA G4s located in gene promoters, mRNA 5′UTR RNA G4s could be responsible for the co-regulation of the expression levels of mRNAs with different functions [105]. Currently, multiple efforts are being driven toward the design and development of ligands that would target G4s in specific mRNA transcripts [106]. Halder et al. reported the interaction of bisquinolinium ligands (PhenDC3, PhenDC6 and 360A) (Figure 6) with RNA G4s present in the 5′UTR *TRF2* mRNA [107]. The driving mechanism for the binding of bisquinolium ligands toward G4s was proposed to be π–π stacking with external G-tetrads [107]. Miglietta et al. identified anthrafurandione derivatives (Figure 6) as potential ligands of 5′UTR *KRAS* G4 [108]. The binding mechanism probably occurs through the tetracyclic cores of ligands that promote π–π stacking interactions with G4 tetrads, whereas the cationic side chains bind to grooves and loops via electrostatic interactions [108].

Emerging evidence indicates that telomeric repeat-containing RNAs (TERRA) actively participate in the mechanisms regulating telomere maintenance and chromosome end protection. In light of this evidence, several ligands have been developed to target TERRA G4s. The interaction of the polyaromatic molecule, RGB-1 (Figure 6), with TERRA and *NRAS* mRNA G4s has been described [109]. The authors proposed that RGB-1 could selectively recognize RNA G4s due to hydrogen bonding acceptors that interact with 2′-OH group of the RNA G4. However, a more deeply structural analysis of the complex RGB-1/TERRA RNA G4 is necessary to understand the binding mechanisms that drive the strong binding affinity and selectivity for RNA over DNA G4s. Carboxy-PDS (c-PDS) (Figure 2) is recognized by strongly exhibit high specificity for RNA [110]. In a study by Rocca et al., they found that c-PDS established π–π stacking interactions with external TERRA G4 tetrads, and several hydrogen bonds with guanine residues [111]. Furthermore, this ligand showed a noteworthy stabilizing effect on TERRA RNA G4 (Δ*T_m_* = 20.7 °C), and the stabilization was not affected by the addition of up to 100 equivalents of a DNA G4 competitor [36]. c-PDS has been successfully applied for the selective stabilization of endogenous RNA G4s in cells [36].

**Figure 7:**
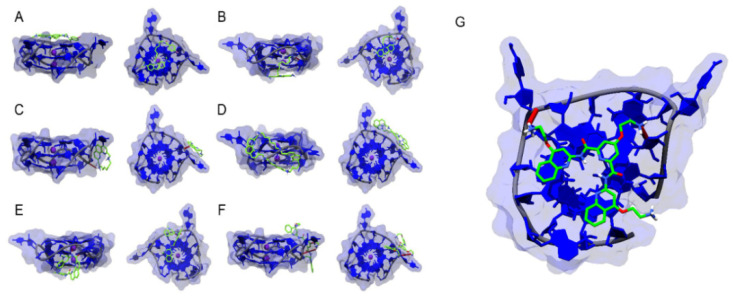
Computational models showing the lowest energy binding poses of the complexes between the prediction model of RNA G4 formed into pre-miRNA-149 and the ligands (**A**) C_8_, (**B**) C_8_-NH_2_, (**C**) (16)phenN_2_, (**D**) (32)phen_2_N_4_, (**E**) PhenDC3 and (**F**) PDS. (**G**) Alternative binding pose of the complex formed by pre-miRNA 149 and PDS after 100 ns of MD simulations. G4 structure is depicted in blue, while ligands are highlighted in green and red. Reprinted with permission from ref. [115] Copyright 2020 Taylor & Francis Group.

Meanwhile, various G4 ligands were computationally and experimentally investigated for their ability to interact with pre-miRNA G4 structures, which have been previously described as key regulatory agents of miRNA biogenesis. Kwok et al. developed a distinguished experimental approach, which they designated SHALiPE, to detect and structurally map RNA G4s at single-nucleotide resolution. The approach is derived from SHAPE method and consists of the coupling of selective 2′-hydroxyl acylation with a lithium ion-based primer extension [112]. They found that pre-miRNA-149 was able to adopt a G4 form in the presence of PDS, which regulates the miRNA-149 biogenesis. Ghosh et al. showed that in the presence of TMPyP4, the pre-miRNA-149 G4 structure was disrupted, leading to a restoration of miRNA-149 biogenesis [102]. The RNA G4 region of pre-miRNA-149 and pre-miRNA-92b were recently characterized, and their interaction with acridine orange derivatives was evaluated [113,114]. Further, six G4 ligands, which have the potential to target the RNA G4 in pre-miR-149 were investigated by means of molecular docking and molecular modelling (Figure 7) [115]. The results suggest that large aromatic surfaces with smaller positively charged side chains capable of performing mixed-mode interactions are preferred on the targeting of RNA G4 formed in pre-miR-149 [115].

**Figure 8:**
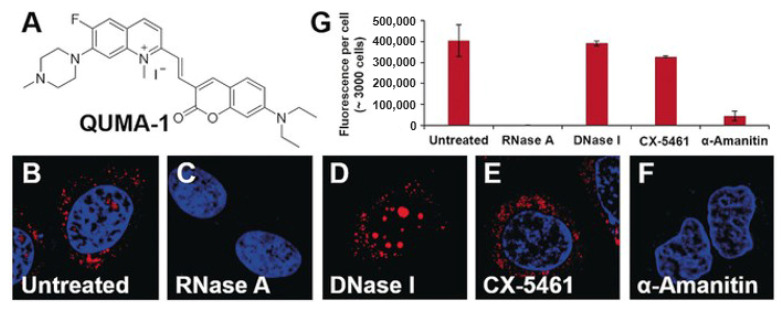
(**A**) Chemical structure of QUMA-1; confocal microscopy images of fixed HeLa cells (**B**) stained with QUMA-1; (**C**) stained with QUMA-1 and treated with RNase A; (**D**) stained with QUMA-1 and treated with DNase I; (**E**) stained with QUMA-1 and treated with CX-5461 and (**F**) stained with QUMA-1 and treated with α-Amanitin. (**G**) Fluorescence intensity per cell. Copyright (2018) Wiley. Adapted with permission from [116] Copyright 2018 John Wiley and Sons, Inc.

As in DNA G4s, a significant number of optical probes targeting RNA G4s have been synthesized in recent years [104]. Chen et al. developed QUMA-1 (Figure 8A), a highly selective fluorescence light-on probe, for tracking and monitoring the folding and unfolding of RNA G4s in live cells (Figure 8) [116]. The interaction between QUMA-1 and RNA G4s was demonstrated to be caused by a rotational restriction that reorganizes the ligand conformation [116].

**Figure 9:**
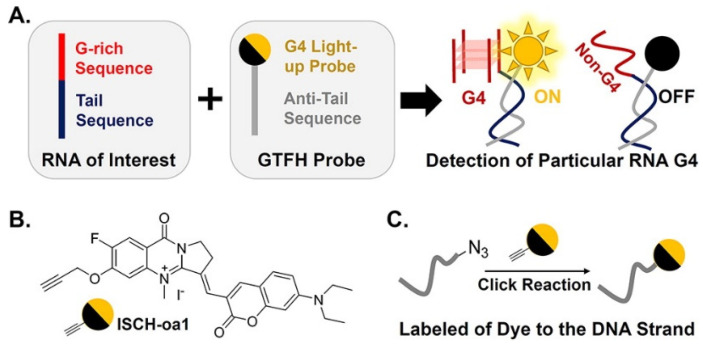
Schematic representation of GTFH probe. (**A**) GTFH probe design principle. (**B**) Chemical structure of ISCH-oa1. (**C**) GTFH probe preparation. Reprinted with permission from ref. [117]. Copyright 2016 American Chemical Society.

Remarkably, the same research group employed more sophisticated engineered approaches to detect G4s in *NRAS* mRNA, with the development of the molecular probe ISCH-oa1 (Figure 9) [117]. They combined the G4 light-on ligand ISCH-1 with an oligonucleotide complementary to the adjacent sequence of the *NRAS* G4 sequence to form an ISCH-*nras*1 ligand that can selectively bind a specific region in *NRAS* mRNA (G4-triggered fluorogenic hybridization (GTFH) probe) (Figure 9) [117]. This approach of sequence-specific recognition of *NRAS* mRNA adjacent to an intended G4 provides a gain of specificity in the transcriptomic context.

**Figure 10:**
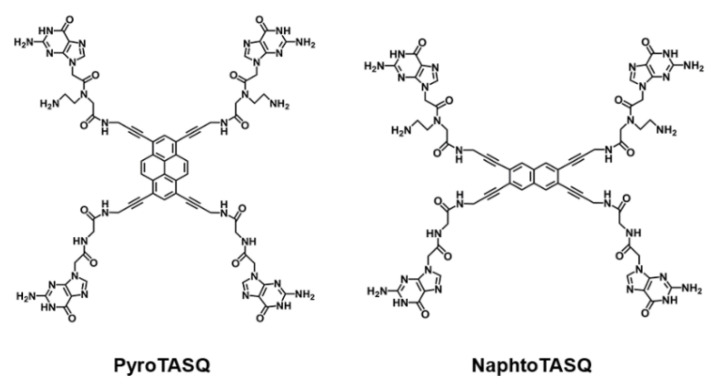
Chemical structures of PyroTASQ and NaphtoTASQ.

Laguerre et al. also described a new multifunctional compound called PyroTASQ (Figure 10), which belongs to both ligands and fluorescent probes [118]. The ligand was designed using the template-assembled synthetic G-quartets method. This multifunctional compound binds to both DNA and RNA G4s in a mechanism that promotes the reorganization of the ligand in a structure that leads to the release of fluorescence by the pyrene moiety [118]. However, in vivo application of PyroTASQ is limited due to its aggregation in cells. Later, in order to overcome this drawback, the same research group substituted the pyrene moiety with naphthalene [119]. The new compound was named NaphtoTASQ (Figure 10) and it was used to visualize DNA and RNA G4s in living cells [120].

## 3. Methods to Characterize G4/Ligand Interactions

In the past few years, the progress in the design and development of numerous methods has helped researchers to assess the binding modes and interactions between G4s and their ligands. These approaches could be different in their nature and more straightforward or complex, but all of them give important information about binding interactions between G4s and their ligands. Many of the methods earlier used to characterize dsDNA interactions have been employed and modified to highlight the contact points between G4s and their binders [121,122]. Biophysical techniques are usually the primary choice in the study of physical interactions and can be divided into three main categories: structure-based methods; affinity and apparent affinity-based methods; and high-throughput methods. In this review, we highlight in detail structure-based methods (circular dichroism (CD), nuclear magnetic resonance (NMR) and X-ray crystallography); affinity and apparent affinity-based methods (surface plasmon resonance (SPR), isothermal titration calorimetry (ITC) and mass spectrometry (MS)); and high-throughput methods (fluorescence resonance energy transfer (FRET)-melting, fluorescence intercalator displacement (G4-FID), affinity chromatography and microarrays). Each method has advantages and drawbacks associated with its features and usually, several methods are necessary to achieve complementary information about G4/ligand interaction [121,122]. This review section discusses biophysical techniques and how they are important to characterize in vitro G4/ligand interactions without cellular relationship.

### 3.1. Structure-Based Methods to Investigate G4/Ligand Interactions

#### 3.1.1. Circular Dichroism (CD)

CD is a standard biophysical method for evaluating the secondary structure, folding, and binding properties of chiral molecules. The method is based on the difference in absorbance by a substance of right- and left-handed circularly polarized light. The intrinsic features of CD make it a powerful tool for the study of the secondary structures and conformations adopted by nucleic acids [123]. The CD spectral signature of nucleic acids arises from (i) asymmetric backbone sugars; (ii) helicity of the secondary nucleic acid structures and (iii) long-range tertiary ordering of nucleic acids in some specific solution conditions [123]. Those important features make CD a method of choice for the study of G4 structures. CD spectroscopy is commonly used to characterize G4 topology, cation effect, G4/ligand interactions and ligand-induced thermal stabilization [124].

**Figure 11:**
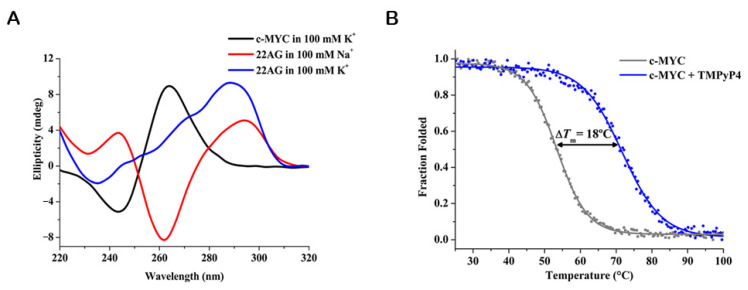
(**A**) CD spectra of G4 structures: c-MYC (10 μM) in 100 mM KCl (parallel); 22AG (10 μM) in 100 mM NaCl (antiparallel) and 22AG (10 μM) in 100 mM KCl (hybrid). The final volume of each sample in the 1 cm path-length quartz cell was 300 µL. The buffer used for the experiments was 10 mM phosphate buffer pH 7.1. (**B**) CD melting curves for c-MYC G4 (10 μM) in 100 mM LiCl in the absence and presence of 2 molar equivalents of TMPyP4. The ellipticity was monitored at a wavelength of 263 nm. The buffer used for the experiments was 10 mM phosphate buffer pH 7.1. Reprinted with permission from ref. [124] Copyright 2017 American Chemical Society.

By using qualitative rules-of-thumb, CD can be easily applied to distinguish different G4 topologies [124,125]. Therefore, the CD spectral features of a given G4 structure are indicative of its topology: parallel G4s have a positive band ≈ 260 nm and a negative band ≈ 240 nm; antiparallel G4s are characteristic by a positive band at ≈ 295 nm and a negative one at ≈ 260 nm and hybrid G4s depict two positive bands (≈ 295 nm and ≈ 260 nm), and a negative one ≈ 245 nm (Figure 11A) [124,125].

Furthermore, the technique has emerged as a strong tool to characterize the interaction of G4s with ligands, being a low-resolution complement to high-resolution methods. NMR spectroscopy and X-ray crystallography are used for high-resolution structural analysis of G4/ligand interactions but are costly, time-consuming and require a large amount of the sample [124,125,126,127]. CD provides a less detailed analysis of the structure, but requires less of the sample. Further, measurements are always performed in solution and are not limited by the molecular weight or size of a molecule [126,127]. Furthermore, the technique provides a fast method to screen G4/ligand interactions, particularly when the timescale of a particular interaction or orientation is short [127]. These advantages significantly contributed to increase the number of studies that employed the technique to analyze the effect of ligands on G4s.

Some specific G4 ligands showed the ability to induce conformational switches in G4s [6,128]. This feature is of utmost importance since it open-up an important framework to control G4 structure and function [128]. In many cases, the new G4 topology is primarily assigned by CD. One of the first examples of a ligand that induced a switch in G4 topology was the triamino-anthracene derivative, which was first designed to interact with G-tetrads by stacking and with the central ion channel with the amine moieties present in the side-chain [129]. Rodriguez et al. monitored the interaction of the ligand with telo24 G4 via circular dichroism spectrum and found that in a sodium buffer, the ligand changes the G4 topology from antiparallel to parallel in about two hours [129]. Interestingly, a porphyrazine ligand can reverse the equilibrium by favoring the antiparallel topology [129]. However, despite some reported examples of ligands that induced a switch in G4 topology centered mainly on the CD data [130,131,132], care must be taken before attributing a new folding topology based on CD results alone. Recent reports have employed CD as a complementary method to evaluate switches in G4 topology [133,134]. Marchand et al. used NMR spectroscopy and mass spectrometry to prove that 360A, PhenDC3, and PDS switch the G4 folding topology of telo22, telo23, telo24 and telo26 [134]. CD spectroscopy was further employed and revealed an antiparallel G4 structure upon ligand binding [134].

In general, G4 ligands are non-chiral and therefore have no CD signal by themselves in solution [135]. However, upon interaction with G4s, those ligands can eventually acquire an induced CD (ICD) signal through the coupling of electric transition moments of the ligand and G4 bases. The observation of an ICD signal is indicative of G4/ligand interactions, but can also perturb the apparent signature of a given G4 topology [135]. As previously mentioned, in those particular cases care must be taken to avoid mistakes in the interpretation of CD results. Nevertheless, the ICD signal could be used to obtain insights on the mode of interaction and geometry of the binding [135].

Ligands that bind G4s by end-stacking or intercalation display only very weak or even no ICD signals [136]. A new class of 9-O-substituted berberine derivatives has been synthesized and their interactions with several G4 structures are characterized by CD titrations [137]. Moreover, in the same study, they also investigated the orientation of the ligand with G4s by analyzing the ICD signals [137]. The same research group reported the binding modes of a selective fluorescent probe of DNA G4s [138].

In the case of groove binders, larger positive ICD signals are generally observed [139]. Zuffo et al. reported that core-extended naphthalene diimides display intense ICD positive signals in the presence of antiparallel and hybrid G4s [139]. In their study, the ligands do not display a detectable ICD signal in the presence of parallel G4s, suggesting a different binding mode that was hypothesized to be end-stacking [139].

CD is also used to determine the binding stoichiometry of G4/ligand complexes and the folding of the G4 structures induced by the ligands. Recently, Głuszyńska et al. employed CD titrations to probe binding stoichiometries and the effect of a fluorescent carbazole derivative in the folding of G4 structures [140]. However, in these particular cases, CD just provides qualitative information about G4/ligand interactions, being frequently a complement for other methods.

Additionally, almost all reported G4 ligands have been characterized by means of CD-melting experiments. The measurement of the melting temperature of the G4 structure by CD is usually performed at the maximum ellipticity wavelength (260 nm or 295 nm), which decreases upon denaturation (Figure 11B) [124]. Thus, depending on the effect of the ligand, the melting temperature of G4s could decrease or increase. In recent decades, the considerable advantages of using CD-melting to study G4/ligand interactions have made it a method widely used by almost all researchers in the field. Indeed, the method is usually one of the primary choices to evaluate the potential of new ligands to stabilize G4 structures.

#### 3.1.2. Nuclear Magnetic Resonance (NMR)

NMR has proved to be fundamental to study the structural, kinetics and dynamics of G4/ligand complexes. The technique provides information about G4/ligand binding under in vitro conditions. In some specific cases, the conditions were set to mimic cellular media with the addition of molecular crowding agents such as PEG and Ficoll [141], and more recently closer to the conditions observed in the living cells using cytoplasmatic extractions or directly inside living cells such as oocytes [142,143,144]. Using 2D ^1^H–^15^N SOFAST-HMQC type experiments to follow changes in chemical shift and intensities, Salgado et al. demonstrated the assembly of the tetramolecular G4 model d(TG_4_T)_4_, inside living *Xenopus laevis* oocytes [143]. They also showed, for the first time, that is possible to study the interaction of ligands with G4s inside living cells [143]. This study opened a new framework to study G4/ligand interactions under in vivo conditions with atomic resolution. Recently, Krafcikova et al. evaluated DNA/ligand interactions inside the nuclei of living human cells [142]. They tested two model DNA fragments and four ligands and the results revealed a strong influence of environmental conditions on the stability of DNA/ligand complexes, especially for one ligand that presented off-target interactions with genomic DNA and cellular metabolites [142]. Their study proved the suitability of the technique to validate ligands, after an in vitro assessment of promising ligands.

The choice of NMR methods to study G4/ligand complexes depends strongly not only on the exchange regime of the individual molecules and the complex, but as well on the ligand properties such as those related with hydrophobicity and immiscibility in water-based solutions. In most cases the polyaromatic properties of G4 targeting ligands require that stocks are prepared in DMSO and other solvents best adapted for hydrophobic compounds. For the vast majority of reported cases, the NMR experiments probe the ligand interaction based on the chemical shift, peak height and linewidth properties of the observed signals. Those experiments are easy to implement and rapid to acquire and analyze. More elaborate experiments based on polarization transfer between spins systems, such as the nuclear Overhauser effect (NOE) [145], Transferred-NOESY [146], Saturation Transfer Difference (STD) [147] and Water-LOGSY [148] methods require more time and elaborated controls. In addition, the abovementioned methods are usually complemented with indirect NMR studies such as H_2_O/D_2_O exchange-rate measurement or paramagnetic spin labels attached in different parts of the G4. As a first approach, most studies take advantage of G4 imino (Hoogsteen base pairing) region near 10–12.5 ppm for initial sampling of ligands. Nevertheless, precautions should be taken in the case of loose G4-ends and loops that do not present imino signals. The advantage is enormous because the aromatic regions (~7–8.5 ppm) are often crowded by many more nuclei from the G4 and the ligand itself, and a direct 1D-visualization and analysis it is not possible. Nevertheless, the imino regions of G4 guanines provide a direct and clear monitoring system for the formation of a G4 structure and its ligand binding interactions.

A study by Yang’s research group showed, by using 2D NMR experiments (DQF-COSY, NOESY and TOCSY), the interaction modes and dynamic binding of BMVC to *c-MYC* G4 [149]. The authors revealed that upon the addition of lower equivalents of BMVC (0–0.7 equivalents), a new set of imino protons appeared, suggesting the formation of a BMVC/*c-MYC* G4 complex [149]. At 1:1 ratio, the imino region showed only one set of imino protons, suggesting a 1:1 complex formation. After the addition of higher equivalents (>1 equivalent) of BMVC, a second set of peaks appeared, which represents the formation of a second complex [149].

However, for high-resolution structural analysis of the complex G4/ligand, 2D NMR and molecular modeling were combined to unambiguously obtain information to construct the 3D model [150]. In addition, the intermolecular NOE interactions can give substantial evidence of the position of the ligand in the 3D structure of the G4 [151]. There are some chemical moieties that are preferable for analyzing the complex G4/ligand, such as methyl groups once they present strong and individual NOE interactions [152].

**Figure 12:**
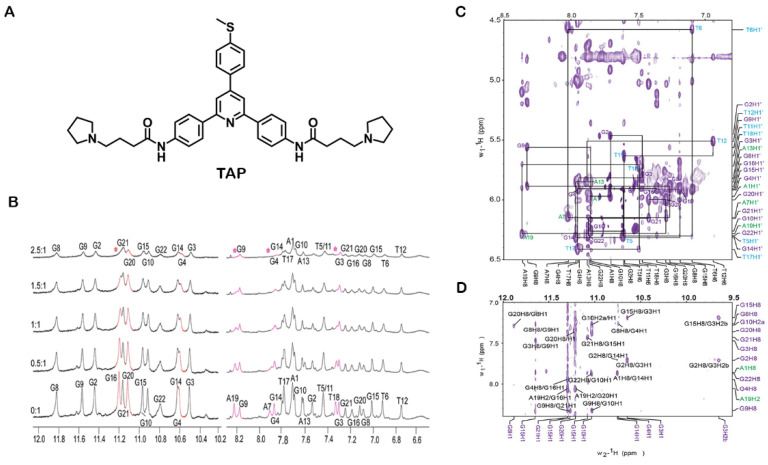
(**A**) Chemical structure of 2,4,6-triarylpyridine (TAP). (**B**) Imino and aromatic proton regions titration spectra of 500 μM 22AG with different concentrations of TAP in 200 μL of sodium phosphate buffer containing 20 mM Na_2_HPO_4_/NaH_2_PO_4_, 70 mM NaCl at pH 6.9. (**C**) Expanded H8/H6-H1′ 300 ms 2D–NOESY spectrum of the 2.5:1 TAP:22AG complex. (**D**) Expanded H1-H8/H2 region from a 300 ms 2D–NOESY spectrum of the TAP:22AG complex at 2.5:1 stoichiometry. Reprinted with permission from ref. [150] Copyright 2017 Elsevier.

Kerkour et al. demonstrated the binding interaction between the G4 ligand, 2,4,6-triarylpyridine (TAP) (Figure 12A), and the 22AG G4 structure (Figure 12B–D) [150]. The ligand presented some signal dispersion indicative of loose binding in multiple conformations. The aromatic protons where not well resolved and the unambiguous restrains used in structural studies originated from the aliphatic protons (Figure 12C). Their NMR data were supplemented with restrained-docking studies to determine the 3D model of the complex TAP-22AG G4. The results obtained with NMR spectroscopy are consistent with those obtained with molecular docking experiments and revealed two binding sites in the 22AG structure, with the most favored site being the lateral loop formed by T17, T18 and A19. In addition, the results depicted a low predisposition of TAP to bind through π–π stacking, preferring the grooves and loops interface.

**Figure 13:**
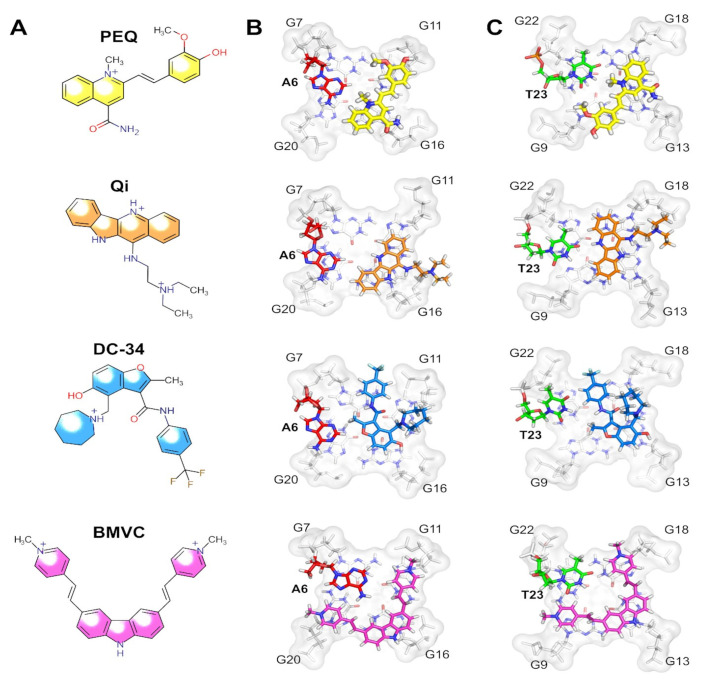
High-resolution structures of Myc2345_T23 in complex with PEQ, Quindoline-I, DC-34 and BMVC. (**A**) Common names and chemical structures of the ligands. Binding of each ligand to the (**B**) 5′-G-tetrad and (**C**) 3′-G-tetrad of Myc2345_T23. Reprinted from ref. [153].

Recently, Yang’s research group reported, by using NOESY, HSQC and DQF-COSY experiments, the NMR solution structures of *c-MYC* complexed with PEQ (Figure 13), a specific *c-MYC* G4 binder with druglike properties [153]. Chemical shift perturbation was used to elucidate macromolecular interactions in the complex G4/PEQ [153]. Furthermore, they have performed a comparison of four available complex structures involving the *Myc*2345_T23 structure and ligands PEQ, BMVC, Quindoline-I (Qi) and DC-34 (Figure 13) [153]. The *Myc*2345_T23 sequence bears a G23-to-T mutation in the 3′-flanking at position 23, in addition to the G14-to-T mutation in the second propeller loop [153]. This structure can be used as a model of parallel G4 structures in promoters and RNA G4s. The same approaches were also implemented to evaluate the complex formation of several G4 ligands various G4 structures [81,154,155,156].

In the last few years, in order to obtain valuable structural information about G4/ligand complexes, Fluorine-19 (^19^F) NMR has become a very useful tool [157]. The advantages of ^19^F NMR spectroscopy include simplicity and sensitivity as well as its ability to study large complexes that cannot easily be probed by conventional NMR experiments [157]. Moreover, it has been applied to study G4 structures, due to the high sensitivity of the ^19^F chemical shift to the environment [158,159].

**Figure 14:**
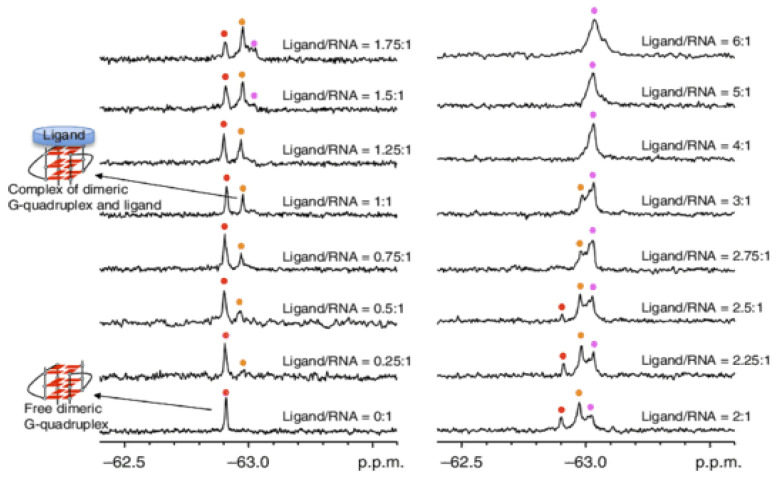
^19^F NMR spectra of ^19^F-labeled 0.5 mM RNA (^19^F-5′-UAGGGUUAGGGU-3′) in 150 µL of 90% H_2_O/10% D_2_O, 10 mM Tris-HCl buffer (pH 7.0), 50 mM KCl. titrated with PDS. Copyright (2017) Wiley. Reprinted with permission from ref. [159] Copyright 2017 John Wiley and Sons, Inc.

Bao et al. demonstrated that ^19^F could be a powerful technique to analyze the interaction of RNA G4s with ligands [159]. They analyzed the complex structure between the fluorinated telomeric RNA G4 (^19^F-5′-UAGGGUUAGGGU-3′) and the well-known G4 ligand PDS (Figure 14). ^19^F NMR enables researchers to distinguish the free and complexed RNA G4, because it is highly dependent on the structural environment of the ^19^F label [159]. Their results revealed that PDS interacts with dimeric G4s but also with high-order G4s with two subunits stacked [159]. Furthermore, ^19^F NMR is an attractive tool to determine *K*_D_ and *T_m_* of the complex RNA G4 and PDS [159]. Binding stoichiometry of PDS to dimeric G4 was 2:1 in a ratio of 6:1 of the ligand to RNA, and the *K*_D_s were 0.63 mM and 1.24 mM for the first and second binding events, respectively [159]. The Δ*T_m_* values of dimeric and high-order G4s were >20 °C and 10.7 °C, respectively [159]. The same research group also demonstrated the utility of ^19^F NMR for the observation and quantitative thermodynamic characterization of telomeric RNA and DNA G4s within in living cells [160,161,162]. Overall, ^19^F NMR offers a suitable and sensible approach to obtain structural insights of DNA and RNA G4s and their complexes with ligands.

#### 3.1.3. X-ray Crystallography

The application of X-ray crystallography toward a structural understanding of G4/ligand complexes enables a detailed structural and spatial characterization of the intermolecular interactions at the atomic level. Furthermore, the considerable advances in X-ray crystallography, such as the miniaturization and automation of crystallization trials, have converted the technique into a method that can be used for screening purposes. One of the advantages of X-ray diffraction over the other techniques is that ligand binding sites can be precisely determined through electron density maps [163]. Furthermore, high-quality X-ray data (i.e., better than 2.5 Å) make the following steps more reliable and allow the visualization and quantification of G4/ligand hydrogen bonding and electrostatic interactions [163]. Moreover, the role of water molecules in the G4/ligand interaction can be determined. However, some important limitations of the technique should be highlighted, such as, (i) the need of a high amount of sample; (ii) the use of cryogenic temperature that can damage artifacts. (iii) the sample must be crystallizable; and iv) it cannot be performed in solution, which limits its application in biological samples. Nevertheless, the technique could be used to obtain detailed information of G4/ligand interactions and has been used in the last year by several researchers in the field.

The earliest reported crystal structures of G4/ligand complexes were the tetramolecular G4/daunomycin complex [164] and the telomeric G4 from Oxytricha nova/acridine derivative complex [165]. Since then, several G4/ligand complexes have been characterized using X-ray crystallography [166,167,168,169].

Recently, Bazzicalupi et al. unveiled the crystal structure of human telomeric DNA G4 complexed with berberine [169]. The results showed that berberine binds toward the G4 telomeric structure in a 2:1 molar ration through π-stacking interactions. The authors stated that strong interaction can be attributable to the coplanarity of the G5/G11 and G17/G23 pairs and to the modified conformation of the 3′-end tetrad.

**Figure 15:**
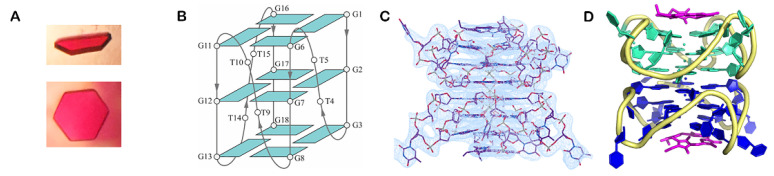
Crystal structure of the T7-NMM complex. (**A**) Representative crystal morphologies: half-hexagonal and hexagonal. (**B**) Schematic representation of the folding topology with the numbering scheme for T1. (**C**) Electron density maps of the T7-NMM dimer. (**D**) Crystal structure of the T7-NMM complex. The T7-NMM sample was prepared by annealing T7 with 1 eq. of NMM at 0.65 mM in 10 mM lithium cacodylate pH 7.2 and 20 mM KCl. Drops were set by the TTP Labtech Mosquito Crystal liquid handler equipped with a humidity chamber at 0.1 μL DNA sample and 0.1 μL of the crystallization condition. Small hexagonal crystals grew within three weeks to 80 μm in the largest dimension from condition C5 of the Natrix screen (Hampton Research): 4.0 M LiCl, 0.01 M MgCl2, and 0.05 M HEPES sodium pH 7.0. Crystals were cryoprotected in the base condition supplemented with 15% ethylene glycol before being flash frozen in liquid nitrogen. Adapted from ref. [166].

Lin et al. investigated the interaction of the G4 forming sequence, 5′-GGGTTGGGTTGGGTTGGG-3′ (T1) and several other sequences based on T1, with the G4 ligand, N-methyl mesoporphyrin IX (NMM) by biophysical and X-ray crystallographic studies [166]. They solved the crystal structure of T1- and 5′-TGGGTTGGGTTGGGTTGGGT-3′ (T7)-NMM complexes. Both structures produced large hexagonal crystals and the T1-NMM crystal structure was determined with a resolution of 2.39 Å, while the T7-NMM crystal structure was solved with a resolution of 2.34 Å (Figure 15). The results showed that both complexes form a 5′-5′ dimer of parallel G4s, which interact with NMM in 3′ G-tetrad through stacking interactions. However, taking into account that one of the main limitations of X-ray crystallography is its non-dynamic nature, they investigated the correlation of the results obtained by X-ray crystallography with techniques performed in solution, namely, CD and PAGE. The T1-NMM complex is concentration-dependent and exists as a mixture of monomer and dimer, whereas the T7-NMM complex only exists as monomer at low and high concentrations. These data suggest that the T7-NMM complex determined by crystallographic studies could be an artifact of the crystal packing.

The most commonly used techniques to structurally characterize, in detail, the G4/ligands interactions are above described and can provide crucial information about structure–function relationships. The major advantages and limitations of each structure-based technique are summarized in Table 1.

### 3.2. Affinity- and Apparent Affinity-Based Methods to Investigate G4/Ligand Interactions

#### 3.2.1. Surface Plasmon Resonance (SPR)

**Figure 16:**
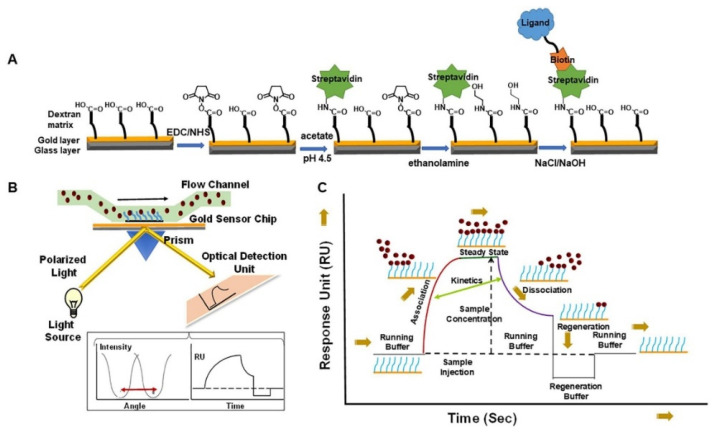
Schematic representation of (**A**) immobilization of biotin labelled RNA G4 on CM5 chip, (**B**) biomolecular recognition in the flow cell with changes in the angle of incidence light and (**C**) SPR sensorgrams. Reprinted with permission from ref. [173] Copyright 2019 Elsevier.

SPR is a label-free powerful method that combines microfluidics and optics to study the interactions of G4s with ligands (Figure 16). The method provides a real-time equilibrium and kinetic characterization for G4 interactions with small amounts of materials and no external probe. The G4 sequence is immobilized on the surface and the ligand is flowed through a microfluidic system. The interaction of the ligand with the G4 is measured by following the changes in the surface refractive index, which modify the angle of the incidence of light. The surface refractive index changes are converted to resonance units (RU) and the results are presented as sensorgrams or binding curves.

The advantages of using SPR are as following: (i) acquisition of data in real-time; (ii) time efficiency; (iii) no need for sample labeling and (iv) high sensitivity, which permits the use of a small amount of the sample [170]. However, some disadvantages have been pointed out to the technique: (i) it requires sophisticated instrumentation and it is costly; (ii) the high dependence on the experimental conditions; (iii) the requirement of maintaining the G4 structure intact after immobilization; and (iv) great care has to be taken in the analysis of the collected data. Indeed, significant efforts have been employed to overcome these issues [170]. The most reliable and suitable immobilization strategy to evaluate the G4/ligand interactions is to have the biotin covalently attached to the 5′-end of the G4 sequence. This strategy ensures the proper assembly of the G4 structure after immobilization and prevents any perturbation that might happen by steric hindrance. However, care must be taken since the addition of biotin covalently attached to 5′-end may impact the G4 conformation, as modifications at the 5′ or 3′ very often do. The use of other complementary experimental techniques must be considered to rule out this hypothesis. Using this immobilization strategy, Perenon et al. used SPR experiments to investigate the interaction between N-methyl mesoporphyrin IX (NMM) and different G4 topologies. They determined the dissociation constant of parallel G4s and showed that it was at least ten times lower than for other topologies [171]. Recently, the binding affinities of the well-known G4 ligands, PhenDC3, 360A and PDS, with the G4 aptamer AS1411 and its derivative AS1411-N6, which have six nucleotides to the 5′-end that are complementary to other six nucleotides at the 3′-end, were assessed by means of SPR [172]. The same strategy of immobilization was used with the oligonucleotides labeled at 5′-end with biotin. The results of SPR measurements are in line with the results obtained by melting experiments [172].

**Figure 17:**
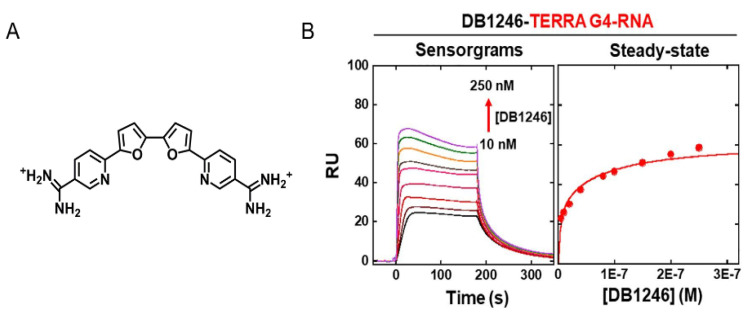
(**A**) Sensorgrams with increasing concentrations of the diamidine derivative DB1246. (**B**) Binding curve showing a two-site binding model with *K*_D1_ = 3 ± 0.8 nM and *K*_D2_ = 80 ± 12 nM. For the immobilization procedure, ~100 µL of 25 nM of TERRA G4 were prepared in HBS-EP buffer (10 mM HEPES pH 7.4, 150 mM NaCl, 3 mM EDTA, 05% *v*/*v* polysorbate 20). The compound DB-1246 (300 µL) was injected in the system from low (10 nM) to high concentration (250 nM). Reprinted with permission from ref. [173] Copyright 2019 Elsevier.

Similar to other techniques, the first SPR studies were carried out to evaluate DNA G4/ligand interactions. However, considering the growing interest in RNA G4s, the SPR studies to assess the interaction of these molecules with ligands have increased [173]. The interaction of diamidine derivative ligands with TERRA was recently reported as an example of using the SPR experiments to assess G4/ligand interactions [173]. The results showed a two-site binding model of diamidine derivative, DB1246, toward TERRA G4 (Figure 17). The binding affinity was in the nanomolar range with *K*_D1_ = 3 ± 0.8 nM and *K*_D2_= 80 ± 12 nM [173]. Tan et al. also reported the use of SPR experiments to determine the binding affinity of miR-3620-5p G4 toward the ligand sanguinarine [174]. They demonstrated the existence of two binding sites for the binding of sanguinarine to miR-3620-5p G4 structure, and revealed that dissociation constants for those binding sites are 0.12 µM and 4.83 µM. In general, SPR provides valuable kinetic and affinity information about G4/ligand complexes.

#### 3.2.2. Isothermal Titration Calorimetry (ITC)

ITC is an experimental method that is especially useful to characterize the thermodynamics of molecular interactions. The technique measures the heat generated or consumed upon the interaction of G4s with ligands [175]. While the three-dimensional structure of a G4/ligand complex may reveal specific interactions, ITC provides insights into the molecular forces that drive the association of G4 with ligands [175,176]. Furthermore, it is a robust technique that measures (Δ*H^0^*), the free energy of Gibbs (Δ*G^0^*) and entropy (Δ*S^0^*) in a single experiment the binding enthalpy, and it is a high-accuracy method for measuring binding affinities and stoichiometry [177].

Recently, Funke et al. investigated via ITC the thermodynamic parameters of the binding of indoloquinoline derivatives to G4s located at *c-MYC* promoter region [178,179,180]. The binding of a ligand to a G4 structure in multiple sites is frequently reported; however, in their recent study, they reported a less common phenomenon that involves the observation of a 2:1 G4/ligand association [180]. Those findings could be explored to control the formation of G4 aggregates.

**Figure 18:**
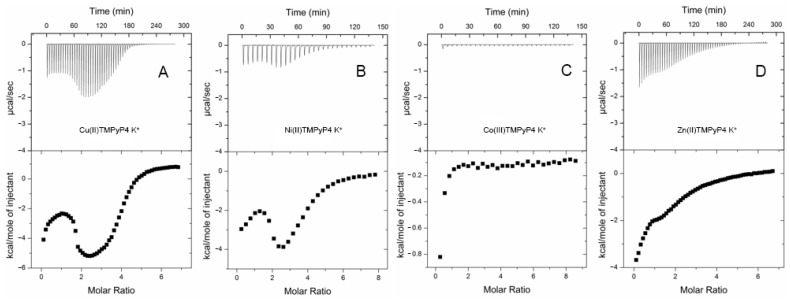
Representative ITC titration profiles for the titration experiments between hTel22 and (**A**) Cu(II)-TMPyP4, (**B**) Ni(II)-TMPyP4, (**C**) Co(III)-TMPyP4, and (**D**) Zn(II)-TMPyP4 in 150 mM K^+^ BPES buffer. ITC experiments were performed of either 28 (10 μL) or 56 (5 μL) injections of a dilute ligand solution into a dilute hTel22 DNA solution. Adapted with permission from ref. [182] Copyright 2016 Elsevier.

The isotherms profile of the interaction of TMPyP4 with telomeric G4s was characterized by two well-differentiated binding events [63,181]. Dupont et al. studied the binding of TMPyP4 and its metalated complexes (Co(III), Ni(II), Cu(II), and Zn(II)) toward the 22-mer oligonucleotide of the telomeric G4 [182]. Different metal center coordination geometries showed distinct thermodynamic signatures (Figure 18) [182]. Their results also showed that TMPyP4, Ni(II)-TMPyP4, and Cu(II)-TMPyP4 bind tightly to G4 structures through an end-stacking/intercalation mode, whereas Co(III)-TMPyP4 and Zn(II)-TMPyP4 bind poorly to the G4 structure in a mechanism consistent with end-stacking.

A comparative thermodynamic study carried out by Bončina et al. showed a different thermodynamic profile for the binding of ligands with low G4 selectivity over duplexes and ligands highly selective for G4 structures [183,184].

The determination of thermodynamic parameters for RNA G4/ligand interactions is less common and there are only a few reports in the literature. One of those reports characterized the binding of TMPyP4 to DNA and RNA repeats of C9orf72 [185]. For DNA repeats, the ITC experiments revealed that TMPyP4 binds through end-stacking and intercalation, whereas for RNA repeats it binds through two different modes, consistent with groove binding and intercalation.

#### 3.2.3. Mass Spectrometry (MS)

MS is an analytical method that is used to measure the mass-to-charge ratio of ions. The technique is highly sensitive, accurate and versatile in its application and can be used to specifically identify multistranded nucleic acid structures, such as G4s [186,187]. In particular, electrospray ionization mass spectrometry (ESI-MS) has been widely applied to probe G4/ligand interactions [134,186,187]. The main advantage of ESI-MS relies on the preservation of non-covalent interactions in the gas phase. From a single spectrum and with low amounts of sample, it is possible to determine detailed structural information (e.g., formation, binding affinity and stoichiometry) of a G4/ligand complex [187]. Monitoring cation binding by ESI-MS provides useful information into ligand binding modes. The number of bound K^+^ in the free and ligand-bound forms of G4s is indicative of the number of G-quartets formed for each ligand binding stoichiometry [188]. Furthermore, the changes in the number of K^+^ upon ligand binding hint at structural conformational changes [188]. Lecours et al. investigated the interaction of PhenDC3, 360A and PDS with several G4 structures through this approach [188].

Marchand et al. also investigated the interaction of (Cu(ttpy)(NO_3_)_2_) with 21 different G4 sequences by ESI-MS [189]. They obtained evidence for the selective and cooperative 2:1 binding of the complex to telomeric G4s. Their results also showed a preference for antiparallel structures with diagonal loops and/or wide-medium–narrow-medium groove-width order. The cooperative binding comes with a conversion of the G4 structure to an antiparallel conformation with three G-quartets.

Ceschi et al. employed ESI-MS to study the binding of perylene derivatives, PIPER and K20, to *KIT2* G4 [190]. Their results revealed a binding stoichiometry of 1:1 on both ligands. They also demonstrated the incapacity of the ligand to bind the unfolded oligonucleotide [190]. Moreover, the preferential binding of K20 to a two-quartet topology was demonstrated, since the most represented complex was the one having a single selectively coordinated K^+^ ion, while only a small amount of the complex having two K^+^ was detected [190]. On the other hand, PIPER showed a reduced preference for a two-quartet topology since the complexes with one and two K^+^ ions were equally represented [190].

**Figure 19:**
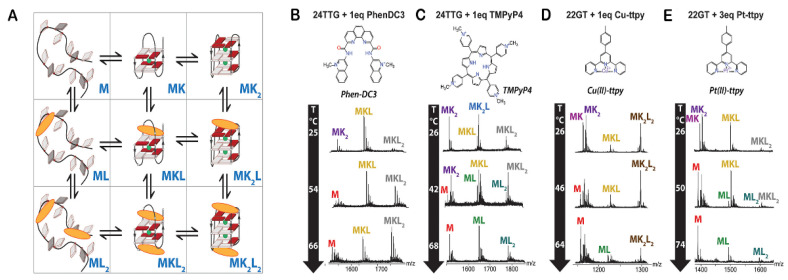
(**A**) Chemical equilibria for ligand binding to a G4 forming sequence. Mass spectra at different temperatures of 10 μM of human telomeric DNA sequence (**B**,**C**: 24TTG; **D**,**E**: 22GT) in the presence of 1 or 3 equivalents of (**B**) Phen-DC3, (**C**) TMPyP4, (**D**) Cu-ttpy, and (**E**) Pt-ttpy. The buffer used was 100 mM TMAA and 1 mM KCl. The volume of oligonucleotide solution was approximately 10 µL. Reprinted with permission from ref. [191] Copyright 2017 American Chemical Society.

Moreover, MS-melting experiments are significantly helpful in explaining inconsistent results between isothermal and melting experiments. The research group of Gabelica used a temperature-controlled nanoelectrospray source to determine the contribution of the thermodynamic parameters to the formation of each stoichiometry G4/ligand [191]. They tested different G4 oligonucleotides in the presence of various G4 ligands such as, PhenDC3, TMPyP4, Cu-ttpy and Pt-ttpy (Figure 19). They reported that ligand stoichiometries differ from ligand to ligand [191]. The ability to distinguish between unfolded, partially folded (two-quartet) and fully folded (three-quartet) ligands is helpful since some ligands have the ability to induce significant changes in topology toward partially folded structures [191].

Recently, the same research group demonstrated the applicability of a top–down mass spectrometry sequencing approach, called electron photodetachment dissociation (EDP), in the study of G4/ligand interactions [192]. They found that the G4 ligands PhenDC3 and 360A can replace a K^+^ ion and bind close to the central loop of telomeric G4 sequences with the ability to form three-tetrad G4s [192].

Scalabrini et al. developed an approach that increases the G4 sensitivity without affecting physiological folding [193]. They optimized the solvent conditions and reported that isopropanol in a triethylamine/hexafluoroisopropanol mixture significantly increases the sensitivity of the technique [193]. The increased sensitivity enables the use of low concentrations of G4s and their ligands. This study could pave the way for future developments of the technique that permit the high-throughput screening of new G4 ligands [193].

Overall, MS is a powerful quantitative technique for characterizing the G4/ligand complexes structurally.

Taking into account that one of the key future challenges of the G4 field still remains in the design and development of ligands that possess selectivity and affinity for DNA or RNA G4s, the techniques here described offer important comparative information on those parameters. The advantages and disadvantages of each affinity and apparent affinity-based methods were summarized in Table 2.

### 3.3. High-Throughput Methods to Investigate G4/Ligand Interactions

#### 3.3.1. FRET-Melting

**Figure 20:**
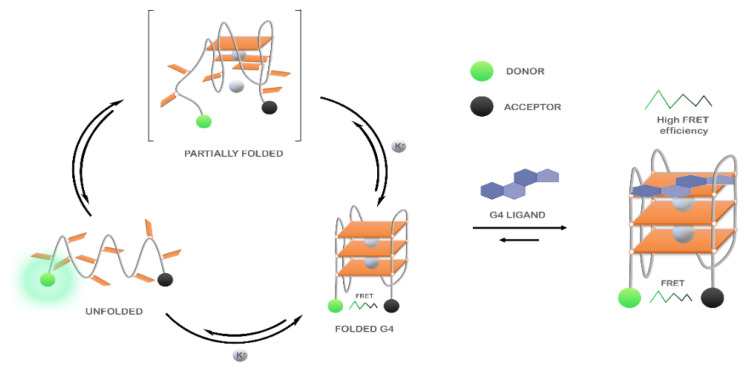
Schematic general representation of FRET-melting experiments.

The Förster resonance energy transfer (FRET) spectroscopy is one of the most used high-throughput methods to study G4/ligand interactions. The technique is highly suitable to evaluate the changes in the G4 folding and unfolding processes (Figure 20). In FRET experiments, G4 oligonucleotides are labeled at the 5′- and 3′-end with a donor and acceptor fluorophore, respectively [194]. Briefly, the excited state energy is transferred from the donor to the acceptor via dipole–dipole interactions. Changes in the distance of the two labeled sites result in a measurable energy transfer. The most common FRET pairs include 6-carboxyfluorescein (FAM) as a donor and 6-carboxy tetramethylrhodamine (TAMRA) or Black Hole Quencher 1 (BHQ1) as acceptors. However, care must be taken since because modifications at 5′ and/or 3′ may alter G4 folding process. Besides that, the method only measures ligand-induced G4 stabilization and does not provide information about other types of interactions.

Despite this, the method has significant advantages and has been widely used to investigate the binding of several classes of G4 ligands, such as phenanthrolines [195], acridines [114], indoloquinolines [196,197] and pyridine-2,6-dicarboxamide derivatives [198] to various G4 structures.

**Figure 21:**
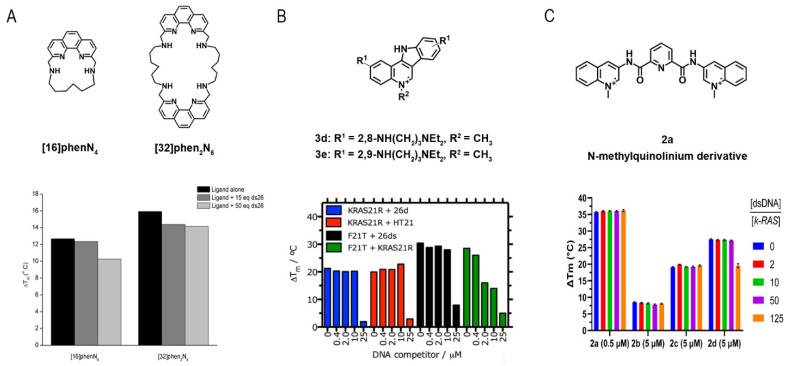
(**A**) Chemical structures of (16)phenN_4_ and (32)phen_2_N_4_ and FRET-melting stabilization by (16)phenN_4_ and (32)phen_2_N_4_ (1 μM) of the labelled F21T sequence (0.2 μM) in buffer 10 mM KCl + 90 mM LiCl and in the presence of increasing concentrations of competitor ds26 (0, 15 and 50 equivalents). Reproduced with permission from [195]. Copyright 2018 Royal Society of Chemistry. (**B**) Chemical structures of indolo(3,2-c)quinoline derivatives **3d** and **3e**, and FRET-melting competition assay with **3e** (1 μM) in complex with *KRAS*21R and F21T G4s (0.2 μM), with increasing concentrations of non-labeled 26ds, HT21 or *KRAS*21R (0.4 to 25 μM) competitor, in cacodylate buffer, pH 7.4 supplemented with K^+^. Reprinted from ref. [197], (**C**) Chemical structure of N-methylquinolinium derivative **2a** and FRET-melting competition assay results for compounds **2a**–**d** at 0.5 μM (**2a**) and 5 μM (**2b**–**d**) complexed with *KRAS* G4 (0.2 μM), challenged with increasing concentrations of non-labeled 26ds DNA (dsDNA) (0.4 μM to 25 μM) competitor. Experiments were performed in cacodylate buffer, pH 7.4, supplemented with 60 mM K^+^. Reprinted from ref. [198].

The ability of four phenanthroline polyazamacrocycles to bind and stabilize G4 structures was assessed by FRET-melting [195]. In this study, the ligand (32)hen_2_N_4_ showed a higher thermal stabilization effect toward c-*MYC* and 22AG G4 sequences at 10 molar equivalents (17.2 °C and 20.3 °C, respectively). The ligands’ selectivity was also evaluated toward other G4s located in oncogene promotors such as *KRAS*-21R, *VEGF* and *c-KIT87*. The results revealed a preference of the ligands toward *KRAS*-21R over *VEGF* and *c-KIT87*. Furthermore, the four ligands demonstrated selectivity and specificity toward G4s over duplex DNA. Indeed, in the presence of 50 molar equivalents of a competitor double-stranded sequence, the stabilizer effect of the ligands (16)phenN_4_ and (32)phen_2_N_4_ in 22AG does not change significantly (Figure 21A).

The stabilization and selectivity of acridine derivatives toward a G4 sequence found in pre-miRNA-92b were recently assessed using FRET-melting [114]. The acridine derivative C_8_, at one molar equivalent, showed the ability to stabilize the structure in 6.3 °C. However, in the presence of 500 nM of RNA-binding domains 1 and 2 of nucleolin, the G4 sequence was stabilized in 22 °C. The remarkable synergistic effect can be attributed to an additional stabilization of the protein by C_8_.

Lavrado et al. also reported the synthesis of novel indolo(3,2-b)quinolines with one, two, or three basic side chains and evaluated its ability to bind and stabilize G4 sequences by FRET-melting [196]. For all the ligands, the thermal stabilization is concentration-dependent. Later, they reported the synthesis of indolo(3,2-c)quinolines and evaluated their interaction toward *KRAS*-21R sequence and a 26-mer hairpin double-stranded DNA sequence (dsDNA) [197]. FRET-melting results revealed that compounds **3d** and **3e** have promising stabilizing effects in *KRAS*-21R (19.9 °C and 22.0 °C, respectively) sequence but not in the dsDNA (5.6 °C and 6.5 °C, respectively). They also reported the selectivity of the compound **3e** in the presence of increasing concentrations of different non-fluorescent competitors (26mer ds-DNA (ds26) or the human telomeric sequence (HT21)). The results revealed a remarkable selectivity of the compound **3e** towards *KRAS*-21R (Figure 21B).

In a recent study, Cadoni et al. reported the synthesis of Pyridine-2,6-dicarboxamide derivatives and evaluated their ability to stabilize G4 structures using FRET-melting [198]. Their results revealed that all the N-methylquinolinium derivatives displayed a preference for G4 sequences when compared to a duplex DNA sequence (T-loop). Moreover, the results showed a noteworthy ability of compound **2a** to stabilize G4 structures even at lower concentrations of the ligand. In fact, at 2.5 molar equivalents of **2a**, the sequence of the *KRAS* G4 was stabilized in 32.8 ± 0.2 °C. They also evaluated the selectivity of the methylated compounds in the presence of double-stranded DNA. The data showed a neglectable effect of the competitor in the G4/ligand complex, which proved the high selectivity of the ligands toward G4 structures (Figure 21C).

In the last few years, some modifications of the method have been carried out to improve the data output and surpass the early limitations of the technique.

A modified transition-FRET method was employed by Noureini et al. to estimate the selectivity of isoquinoline alkaloids from *Chelidomium majus* toward a human telomeric G4 [199]. The novel FRET method consists of two transition steps: the first transition step is the melting temperature of the double helix structure, whereas the second transition state belongs to G4 denaturation. Therefore, in the presence of selective G4 ligands, an increase in the melting temperature of the second step is observed, as well as a decrease in the fluorescence intensity of the first step.

Rakers et al. developed a novel microfluidic platform to synthesize novel G4 binders and evaluate their affinity and using a real-time online FRET assay [200]. Furthermore, their strategy enabled the high-throughput online analysis of several G4 binders. Thus, this study could trigger novel strategies that combine the synthesis and the assessment of G4 binders in a unique device.

An important feature of FRET-melting is its remarkable competitive nature [201], when compared with the other techniques mentioned in this review. Indeed, most of the techniques used to investigate G4/ligand interactions are mainly comparative, which makes it a strong competitive tool to analyze selectivity and specificity.

In a very recent report, Luo et al. also explored the competitive nature of FRET-melting and studied whether the interaction between a fluorescent G4-forming oligonucleotide and PhenDC3 is modified by a competitor sequence added in excess [202]. Sixty-five sequences with a known structure were tested to validate this FRET-melting competition (FRET-MC) assay. In this case, if the unlabeled competitor has a high affinity for PhenDC3, it will sequester a significant fraction of the compound, which will be no longer available for fluorescent G4-forming oligonucleotide stabilization, leading to a decrease in *T*_m_. On the other hand, if the competitor sequence is unable to sequester the ligand, *T*_m_ remains high.

#### 3.3.2. G4-FID Screening

The fluorescence intercalator displacement (FID) assay is based on the competitive displacement of thiazole orange (TO) fluorescent probe from various G4 and duplex by increasing amounts of the ligand subjected to evaluation.

**Figure 22:**
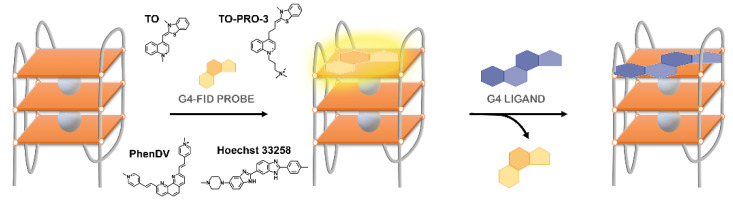
Schematic general representation of G4-FID experiments. Chemical structures of TO, TO-PRO-3, Hoechst 33258 and PhenDV.

Monchaud et al. have optimized the original FID assay to study G4/ligand complexes [203,204,205]. The technique does not require modified oligonucleotides and it is based on the loss of fluorescence of the bound intercalator, thiazole orange (TO), upon displacement induced by a G4 ligand (Figure 22).

**Figure 23:**
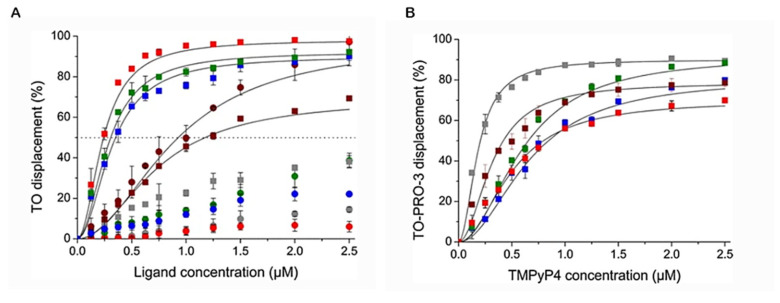
(**A**) Representative G4-FID curves obtained with MMQ1 (gray), MMQ3 (brown), Pt-ttpy (red), 360A (blue) and Phen-DC3 (green) with 22AG.K (squares) and ds26 (circles) using TO as fluorescent probe. (**B**) Representative G4-FID curves of TMPyP4 with 22AG.Na (blue), 22AG.K (red), c-kit1 (brown), c-kit2 (gray), and ds26 (green) with TO-PRO-3 as fluorescent probe. For each well of the microplate were added: (i) K^+^- or Na^+^-buffer solution; (ii) oligonucleotides (5 μM) and fluorescent probe (TO or TO-PRO-3; 10 μM for G4-DNA or 15 μM for ds26) and (iii) an extemporaneously prepared 5 μM ligand solution in K^+^- or Na^+^-buffer. The final volume in each well was 200 µL. Reprinted with permission from ref. [206] Copyright 2011 Springer Nature.

However, the traditional method had some limitations that were correlated with its applicability. To surpass those drawbacks, Largy et al. developed the high-throughput version of the G4-FID assay by analyzing an extensive set of G4 ligands in a 96-well plate (Figure 23) [206]. Further, the spectral range of the assay was expanded by using two other fluorescent probes, Hoechst 33258 and TO-PRO-3, a minor groove binder that emits blue fluorescence and an intercalator that emits red fluorescence, respectively [206]. These fluorescent light-up probes emit low fluorescence when free in a solution, but present a 1000-fold increase when complexed with DNA. The binding affinity of several ligands toward a significant number of DNA/RNA sequences was assessed by G4-FID assay [207]. However, considering the modest selectivity of the fluorescent probes for DNA G4s over duplex DNA and their ambiguous binding mode, alternative dyes have been proposed. PhenDV-based is a bisvinylpyridinium dye that would bind to DNA G4s in a similar mode to that of PhenDC3 [208]. The readout was carried out by fluorescence enhancement and not by quenching [208].

The same strategy of fluorescence enhancement was employed by using the dimeric cyanine small molecule, B6,5, which can be used as a probe in fluorescence displacement assays to screen G4 specific ligands for DNA and RNA G4s [209].

The methods used for processing a huge quantity of data are often ineffective for easily visualizing the binding selectivity of G4 ligands. Villar-Guerra et al. described a rapid FID (R-FID) assay which, when combined with a novel application of principal component analysis (PCA) and hierarchical cluster analysis (HCA), represents a powerful tool for the analysis of large FID data sets [210].

Most of the efforts to improve the G4-FID experiments have been focused on developing novel highly fluorescent probes that could be more selective and specific than the existing alternatives.

#### 3.3.3. Affinity Chromatography Screening

**Figure 24:**
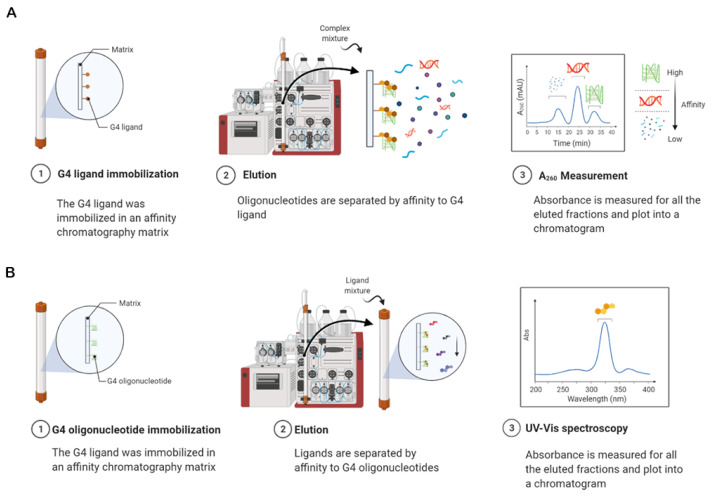
(**A**) Scheme of affinity chromatography strategy to purify G4 sequences based on a ligand affinity chromatography support. (**B**) Scheme of affinity chromatography strategy to purify G4 ligands based on a G4 affinity chromatography support.

Affinity chromatography is a highly effective technique that enables the selective isolation of target molecules [211]. The method relies on selective and reversible interactions that undergo with the binding agent and the target. Thus, the release of the target occurs under specific conditions, where it does not have an opportunity to rebind, and according to its binding affinity toward the recognition agent. Nowadays, the technique has been used in a wide variety of applications across different fields, which include the isolation of G4s from complex mixtures (plasma, serum) [212] (Figure 24A) and the identification of G4 ligands [213] (Figure 24B).

The G4 ligand, N-methyl mesoporphyrin IX (NMM) was coupled to a Sepharose matrix and demonstrated the ability to selectively bind DNA (albeit with distinct binding affinities for different topologies) [212]. This work demonstrated the feasibility and applicability of the method but is somewhat limited by the incomplete recovery of the bound DNA, the use of a ligand with a lack of selectivity for G4 structures, and the employment of batch affinity adsorption rather than column affinity chromatography. Later, the perylene derivative ligand (*N*,*N*′-Bis-(2-(amino)ethyl)-3,4,9,10-perylenetetracarboxylic acid diimide, Pery01) was coupled to a matrix of polyglycidylmethacrylate (PGMA) to isolate DNA G4s by column affinity chromatography [214]. The support displays a preference for parallel G4s and demonstrated capability to isolate parallel G4s from plasma. Ferreira et al., by using the same column affinity chromatography strategy, functionalized a matrix of Sepharose CL-6B with a new naphthalene derivative (L1) and the ability of the support to selectively bind to different G4 sequences was evaluated [213]. The results showed a remarkable binding affinity for parallel G4s over non-parallel G4s as well as other conformations.

**Figure 25:**
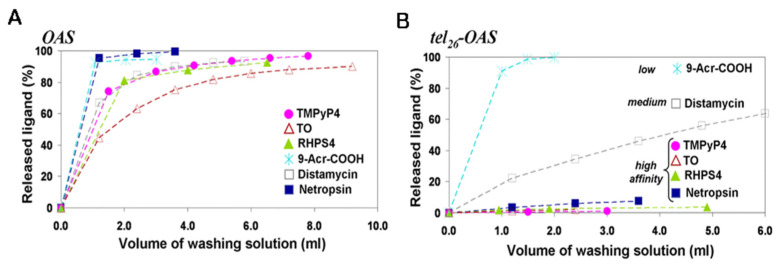
Amount of the released ligands, expressed as percentage of the quantity loaded (**A**) on OAS support and (**B**) on tel26-OAS support, as a function of the washing solution volume (100 mM KCl/15% DMSO). Reprinted from ref. [215].

Musumeci et al. developed an identical approach, named G4 on Oligo Affinity Support (G4-OAS) assay [215]. However, instead of flowing throughout the column a complex mixture containing G4-forming sequences, the method consists of flowing solutions of probable G4 binders through a polystyrene resin functionalized with a G4-forming sequence. The compounds having a high affinity for the G4 sequence are retained in the support, whereas those with no or low affinity are eluted first and quantified by UV measurements (Figure 25). The method is simple and rapid, but there are some important limitations to point out: (i) the unspecific binding of the ligand to the resin and (ii) the absence of structural information about G4 conformation. The high-throughput nature of the method was proved later by using the virtual screening and the G4-OAS assay in tandem [216]. Recently, to overcome those initial limitations, the authors developed a novel functionalization method by employing an approach of solid-phase synthesis of the oligonucleotides on Controlled Pore Glass (CPG) support [217]. The strategy was further employed to analyze a set of naphthalene diimide derivatives by using the sequence d[AGGG(TTAGGG)7] (tel46), which folds into two consecutive G4s [218]. The G4-CPG screening identified the ligand NDI-5 as the most notable ligand of the tel46 sequence.

#### 3.3.4. Microarrays-Based Screening

Microarrays are a fast, simple, and high-throughput method that could be employed to screen ligand interactions with DNA and RNA sequences. The well-known G4 ligand PDS has recently been fluorescently labeled and evaluated toward a hundred thousand G4 sequences, which covalently attached to the surface of glass slides [219]. The binding selectivity of the G4 ligand BMVC to several G4 structures was also assessed by a custom G4 microarrays platform and revealed a preference for parallel G4s [220].

The reverse strategy was employed in small-molecule microarrays (SMMs). The technology relies on the immobilization of the ligands by covalent interaction followed by incubation with the fluorescently labeled oligonucleotide. Although the method has been broadly used to identify protein ligands, it has only recently been transposed to analyze G4/ligand interactions. Felsenstein et al. employed SMMs for the first time in the G4 field and identified a benzofuran derivative as a specific *c-MYC* G4 binder among 20,000 compounds [221]. This initial study has led to the optimization of the ligand in a more potent analog (DC-34), which showed interesting in vivo potency and pharmacological properties in cancers expressing *c-MYC* [222].

High throughput screening is an essential strategy of early-stage drug discovery, and methods and technologies have seen many improvements and innovations over recent years. In many cases, the early high-throughput methods have been optimized and recently, some improvements have been reported in the characterization of G4/ligand interactions. The advantages and disadvantages of the high-throughput methods described in this review are summarized in Table 3.

In addition to the techniques here approached and used for investigating G4/ligand interactions, other robust and efficient biophysical, biochemical and molecular biology techniques are available to describe G4/ligand interactions, such as surface-enhanced Raman spectroscopy [223], single-molecule fluorescence imaging [97,224], equilibrium dialysis [225], gel electrophoresis [114,226], qPCR-stop assay [227], Taq polymerase stop assay [228] and TRAP assay [229]. Furthermore, other high-throughput methods are emerging, such as pull-down assays [230] and Affinity Selection-Mass Spectrometry (ALIS) [231]. These are not described in detail in this review but, depending on the applicability, could be employed to evaluate the molecular interactions between G4s and ligands.

## 4. Conclusions

G4s are recognized as important structural motifs in a wide range of biological processes. Over the past two/three decades, G4s have been intensively studied in the presence of a diverse family of ligands. As a result of these studies, important atomic-detailed information concerning the principal forces governing G4/ligand interactions was revealed. In addition, the results also point out that fundamental chemical aspects remain to be fine-tuned concerning the design and development of ligands that possess selectivity and affinity for DNA or RNA G4s.

From a structural point of view, CD, NMR and X-ray crystallography are techniques with many strengths, with individual limitations rather well complemented among them.

The simple and fast nature of CD makes it one of the primary choices to obtain low-resolution information about structural details. CD is commonly used at an initial stage of the G4/ligand interaction characterization. Users can easily obtain information about the G4 topology and often preliminary information on the binding modes of the ligands. Furthermore, almost all the studied G4 ligands were investigated in terms of their ability to stabilize G4s by CD-melting experiments. Despite the valuable information provided by CD, the full structural characterization of a given G4/ligand complex can only be achieved by using high-resolution structural methods such as NMR or X-ray Crystallography.

For instance, NMR is a solution-based technique that can analyze the dynamics of the complexes on a broad range of time scale (ps-s), whereas X-ray crystallography provides insight into the most thermodynamically stable form of a complex. Under some circumstances using both may be the best option, as some ligands tend to induce aggregates that precipitate in the NMR tube over time and resolution is lost. For some polymorphic oligonucleotides the other way around is preferable, and for those cases where both techniques can be used, they represent the strongest solution to investigate G4/ligand complexes at atomic detail. However, when compared to CD, both NMR and X-ray crystallography have the disadvantage that they often require milligram amounts of material. In the last decade, the lower concentration limit in NMR has been significantly reduced due to the combination of increased magnetic field strength and other sensitivity enhancement technologies, so we expect that the determining of the high-resolution structure of G4s and complexes will continue to be a very active field of research.

Joint analysis using high-resolution NMR and X-ray studies have provided invaluable structural insights into the interaction and binding modes of G4/ligand complexes in the biological context. These structural studies have shown the atomic details of the interaction between G4s and their ligands, allowing researchers to analyze structure–function relationships to rationally design and develop new specific and selective G4 ligands to treat a variety of diseases. However, these structural techniques are not sufficient to characterize completely the driving forces of a given interaction. Indeed, one key challenge still remaining to be addressed is the design and development of ligands that possess selectivity and affinity for DNA or RNA G4s. Therefore, other affinity- and apparent affinity-based methods such as SPR, ITC and MS have been intensively used in the G4 field.

SPR was shown to be a preferable choice in comparison to other in vitro techniques such as ELISA, which does not allow an absolute measurement of kinetic constants, is less sensitive and more expensive in terms of required samples and time. However, the SPR technology requires that one of the analyzed molecules is immobilized on a functionalized metal surface and this often leads to the heterogeneous orientation of the ligand molecule and the alteration of its activity, and a lot of parameters must be carefully controlled, increasing the difficulty of the analysis of the collected data.

Until now, ITC is the only available experimental method to directly measure binding enthalpies without any chemical modification or immobilization of the interacting components. Besides that, ITC also provides a complete characterization of the thermodynamic parameters of G4/ligand interactions.

MS is a powerful technique that provides a direct readout of the stoichiometries of G4/ligands complexes versus duplex formed, including minor species, and it is a very attractive characteristic of this technique, as well as which it does not require modified oligonucleotides and allows using a broad variety of G4s versus duplex. However, electrospray is incompatible with the presence of alkaline salts such as NaCl or KCl and must be replaced by ammonium acetate, entailing changes in the G4 structures and consequently in the binding of ligands. ITC and MS, as well as SPR, require specific know-how and relatively expensive equipment.

The newly emerged high-throughput methods combined with other biophysical approaches created a new framework, which allows the fast and cheap development and characterization of G4/ligand complexes.

For instance, FRET-melting, which is carried out in presence of a competitive duplex sequence, has been adapted for an overwhelming number of G4 ligands. This method is rapid and convenient but requires modified oligonucleotides, and possible interferences between ligands and the fluorescent labels represent the two main limitations of FRET-melting. A suitable alternative/complementary method of FRET-melting is G4-FID, which allows the screening of an important number of unmodified G4 structures with wide-spread equipment i.e., a qPCR devices, fluorimeters or microplate readers.

Affinity chromatography emerged as a simple and fast method to analyze the interaction of ligands with G4s. The method relies on simple and efficient assays by immobilizing G4s on a solid support and is particularly useful to select G4 ligands with specificity and selectivity for a given G4 structure. Further, the method could be used to separate those G4 ligands from a complex mixture. The reverse strategy, in which the ligand is immobilized, could also be employed to separate G4s from complex biological samples.

Microarrays have only recently been used to characterize G4/ligand interactions and few reports are as yet available in the literature. The method is fast and straightforward and can be used to analyze a huge quantity of G4 structures, ligands and conditions. However, its use as a routine technique to analyze G4/ligand interactions could be hindered due to its high cost and need for specialized equipment.

In general, the modification of low-throughput methods to became high-throughput could significantly impact the field. Such progress will naturally occur and lead to the characterization of a great number of G4/ligand complexes.

One of the main challenges of the field is that, at the moment, despite the significant advances in recent years, none of the techniques provides a full characterization of G4/ligand complexes. The complete framework is only achieved with the use of several techniques, which can sometimes be difficult to assemble in a single research institution.

Overall, this review intends to improve the knowledge and understanding of the currently developed methods used to assess G4/ligand interactions, and at the same time provide a broad framework of the chemical features of the G4 ligands available for diagnostic and therapeutic purposes.

## Figures and Tables

**Table 1:** Advantages and limitations of structural-based methods for investigating G4/ligand interactions.

Method	Advantages	Limitations
CD	Simplicity	Low-resolution
	Small amount of sample	
	No need of sample labelling	
	Not limited by the molecular weight or size of the molecules	Most of the ligands are non-optically active
	Can easily provide melting temperature curves and global folding changes	
	Most suitable method for finding the polarity of chains	
NMR	Provides atomic-resolution characterization of a G4/ligand complex	High amount of sample
	Detailed pairs of atoms contacts between ligand and receptor	Time-consuming
	Three-dimensional structures in their natural state can be measured in solution	Limited by size or atomic weight
		Need of isotopic labelling
X-ray	Relatively cheap and simple	High amount of sample
	Provides atomic-resolution characterization of a G4/ligand complex	Cryogenic temperature can induce altered contacts
	Provides void electronic areas in the receptor that can be used to improve ligands design	The sample must be crystallizable
	Not limited by size or atomic weight	Only provides static three-dimensional analysis

Legend: Circular Dichroism (CD), Nuclear Magnetic Resonance (NMR) and X-ray Crystallography (X-ray).

**Table 2:** Advantages and limitations of affinity and apparent affinity-based methods for investigating G4/ligand interactions.

Method	Advantages	Limitations
SPR	Acquisition of data in real time	Requires sophisticated instrumentation and it is costly
	Time efficiency	High dependence of experimental conditions
	High sensitivity	SPR often requires labeling with biotin
	Provides valuable kinetic and affinity information (association (*K*_a_ or *K*_on_), dissociation (*K*_d_ or *K*_off_) andequilibrium (*K*_D_) constants)	Requirement of maintaining the G4 structure intact after immobilization
ITC	Provides insights of molecular forces that drive the interaction	High amount of sample
	Provides kinetic and thermodynamically parameters	Ligands should be soluble in water
MS	Provides information on formation, stoichiometry, and binding affinity of G4/ligand complex	Limitations regarding the media

Legend: Surface Plasmon Resonance (SPR), Isothermal Titration Calorimetry (ITC) and Mass Spectrometry (MS).

**Table 3:** Advantages and limitations of high-throughput methods for investigating G4/ligand interactions.

Method	Advantages	Limitations
FRET-melting	Simplicity	Fluorescently labelled oligonucleotides
	Small amount of sample	It only measures ligand-induced G4 stabilization, while other types of interactions are not detected
	Real-time monitoring	
G4-FID	Simplicity	Ambiguous binding mode of used probes
	Small amount of sample	Compatibility of the oligonucleotides with the fluorescent probe
Affinity Chromatography	Selective and reversible interactions that undergo with the ligand and G4	Unspecific binding of the ligand to the resin
	Column chromatography allow real-time monitoring	The absence of structural information about G4 conformation
Microarrays	Small amount of sample	Expensive
	Massive parallel screening	Specialized equipment
		Fluorescently labeled molecules

Legend: Fluorescence Resonance Energy Transfer (FRET) and G4 fluorescent intercalator displacement (G4-FID).

## Data Availability

Data sharing not applicable.
